# Contact-dependent signaling triggers tumor-like proliferation of *CCM3* knockout endothelial cells in co-culture with wild-type cells

**DOI:** 10.1007/s00018-022-04355-6

**Published:** 2022-06-04

**Authors:** Matthias Rath, Konrad Schwefel, Matteo Malinverno, Dariush Skowronek, Alexandra Leopoldi, Robin A. Pilz, Doreen Biedenweg, Sander Bekeschus, Josef M. Penninger, Elisabetta Dejana, Ute Felbor

**Affiliations:** 1 Department of Human Genetics, University Medicine Greifswald and Interfaculty Institute of Genetics and Functional Genomics, University of Greifswald, Fleischmannstraße 43, 17475 Greifswald, Germany; 2 Vascular Biology Unit, FIRC Institute of Molecular Oncology Foundation (IFOM), Milan, Italy; 3 Institute of Molecular Biotechnology of the Austrian Academy of Sciences, Vienna, Austria; 4 Centre for Innovation Competence-Humoral Immune Reactions in Cardiovascular Diseases, University of Greifswald, Greifswald, Germany; 5 ZIK Plasmatis, Leibniz Institute for Plasma Science and Technology (INP), Greifswald, Germany; 6 Department of Medical Genetics, Life Sciences Institute, University of British Columbia, Vancouver, Canada; 7 Department of Immunology, Genetics and Pathology, Uppsala University, Uppsala, Sweden

**Keywords:** Cerebral cavernous malformations, CRISPR/Cas9 genome editing, Tumor-like behavior, NSC59984, RNA sequencing, Co-culture

## Abstract

Cerebral cavernous malformations (CCM) are low-flow vascular lesions prone to cause severe hemorrhage-associated neurological complications. Pathogenic germline variants in *CCM1*, *CCM2,* or *CCM3* can be identified in nearly 100% of CCM patients with a positive family history. In line with the concept that tumor-like mechanisms are involved in CCM formation and growth, we here demonstrate an abnormally increased proliferation rate of CCM3-deficient endothelial cells in co-culture with wild-type cells and in mosaic human iPSC-derived vascular organoids. The observation that NSC59984, an anticancer drug, blocked the abnormal proliferation of mutant endothelial cells further supports this intriguing concept. Fluorescence-activated cell sorting and RNA sequencing revealed that co-culture induces upregulation of proangiogenic chemokine genes in wild-type endothelial cells. Furthermore, genes known to be significantly downregulated in *CCM3*^*−/−*^ endothelial cell mono-cultures were upregulated back to normal levels in co-culture with wild-type cells. These results support the hypothesis that wild-type ECs facilitate the formation of a niche that promotes abnormal proliferation of mutant ECs. Thus, targeting the cancer-like features of CCMs is a promising new direction for drug development.

**Supplementary Information:**

The online version contains supplementary material available at 10.1007/s00018-022-04355-6.

## Introduction

Cerebral cavernous malformations (CCMs), also known as cavernous haemangiomas or cavernomas, account for 10 to 15% of all vascular lesions in the central nervous system [1]. In particular, hereditary CCMs tend to cause significant neurological complications. Unfortunately, therapeutic options are still limited. Neurosurgical resection can be indicated in some cases but there is a substantial risk of early postoperative morbidity for CCMs in the brainstem or other eloquent areas [2].

CCMs can be found in a sporadic and an autosomal dominant form. The latter is associated with pathogenic germline variants in either *CCM1* (aka *KRIT1*), *CCM2,* or *CCM3* (aka *PDCD10*) [3]. Patients with familial CCMs, especially those with a pathogenic *CCM3* variant, often present at a younger age with multiple cavernomas [3–5]. The number of vascular lesions can even increase over time. Despite our growing knowledge on CCM pathogenesis, it remains unclear why CCMs almost exclusively arise in the central nervous system. Global *Ccm3* gene disruption in mice induces embryonic lethality [6]. In acute models of conditional endothelial-specific *Ccm3* inactivation, numerous CCM lesions can be found that are primarily located in the cerebellum. Chronic models lead to fewer but more randomly distributed CCMs [7]. Interestingly, specific *Ccm3* gene knockouts in astrocytes, neural cells, and brain mural cells can also induce CCM-like lesions in mice [8–10].

Genetic and immunohistochemical analyses of human cavernoma tissues have established a Knudsonian two-hit model for familial CCM disease [11–16]. Only when a somatic mutation inactivates the wild-type allele in a cell of a germline mutation carrier, CCM formation is initiated. With sophisticated transgenic *Ccm3* mouse models, recent studies have shed light on the stages of CCM genesis and demonstrated that clonal expansion of mutant endothelial cells (ECs) and recruitment of wild-type or heterozygous ECs trigger early CCM formation and later lesion growth, respectively [17, 18].

By using human-induced pluripotent stem cell (hiPSC)-derived vascular organoids and EC co-cultures, we here demonstrate that cancer-like proliferation of mutant ECs is only triggered by direct contact with wild-type ECs. The fact that the anti-cancer drug NSC59984, a small molecule known to induce constitutive phosphorylation of ERK2 and reactivate p53 signaling in cancer cells [19, 20], blocked the abnormal proliferation of CCM3-deficient ECs reinforced the hypothesis that some features of CCMs are reminiscent of tumorigenesis [17, 18]. Finally, our study reveals that wild-type ECs activate chemokine signaling pathways in co-culture and thus provides insight into the poorly understood interaction of wild-type and mutant ECs.

## Materials and methods

### Cell culture

Constitutive immortalized human umbilical vein endothelial cells (CI-huVECs, HK0, 240615, InSCREENeX, Braunschweig, Germany) [21] were cultured at 37 °C and 5% CO_2_ in endothelial cell growth medium (ECGM, PromoCell, Heidelberg, Germany) supplemented with 10% fetal calf serum (FCS, Thermo Fisher Scientific, Waltham, MA, USA). Clonally expanded CI-huVEC lines with biallelic loss-of-function variants in the first coding exon of *CCM3* have been described before (Online Resource 1, [22, 23]). Pathogenic *TP53* variants in *CCM3*^*−/−*^ CI-huVECs were excluded using the NEXTflex TP53 Amplicon Panel (Bioo Scientific, Austin, TX, USA). *CCM3* mutant and wild-type CI-huVECs were co-cultured in 96-well plates under standard culture conditions with the indicated mutant-to-wild-type ratios. 2500 cells were seeded per well. To investigate the role of laminin-332, 2500 *CCM3*^+/+^ or *CCM3*^−/−^ CI-huVECs were cultured for 6 days on 96-well plates coated with 1 µg/cm^2^ human recombinant laminin-332 by Biolamina (LN332, Sunbyberg, Sweden). Wells were fixed on day 6 with 4% PFA and immunofluorescence staining was performed for laminin (1:50, sc-133178, Santa Cruz Biotechnology, Dallas, TX, USA) with goat anti-mouse, Alexa Fluor 488 secondary antibody (1:200, A-11029, Thermo Fisher Scientific).

The following endogenously tagged human iPSC lines as part of the Allen Cell Collection (Coriell Institute, Camden, NJ, USA) were used in this study: AICS-0036-006 (00003450):WTC-mEGFP-Safe harbor locus (AAVS1)-cl6 and AICS-0054-091 (00007433):WTC-mTagRFPT-CAAX-Safe harbor locus (AAVS1)-cl91. hiPSC lines were maintained at 37 °C and 5% CO_2_ in Essential 8 Flex medium (Thermo Fisher Scientific) on plates coated with growth factor reduced matrigel (Corning Inc., Corning, NY, USA), passaged with 0.5 mM EDTA (Thermo Fisher Scientific), and checked for the expression of the stem cell markers OCT4, SSEA4, SOX2, and TRA-1-60 using the PSC 4-Marker Immunocytochemistry Kit (Thermo Fisher Scientific). For AICS-0036 lines, a goat anti-mouse, Alexa Fluor 555 antibody (1:500, ab150114, Abcam, Cambridge, UK) and a goat anti-rat, Alexa Fluor 555 antibody (1:500, A-21434, Thermo Fisher Scientific) were used as secondary antibodies to stain for SSEA4 and SOX2. For the AICS-0054 line, a goat anti-rabbit, Alexa Fluor 488 antibody (1:500, A-11008, Thermo Fisher Scientific) and a goat anti-mouse, Alexa Fluor 488 antibody (1:500, A-21042, Thermo Fisher Scientific) were used as secondary antibodies to stain for OCT-4 and TRA-1-60. The hPSC Genetic Analysis Kit (Stemcell Technologies) was used according to the manufacturer's instructions to exclude common chromosomal abnormalities. iPSC cultures were regularly tested negative for mycoplasma contamination by PCR.

### CRISPR/Cas9 genome editing in hiPSCs

For ribonucleoprotein (RNP)-mediated genome editing, hiPSC lines were transfected with sgRNA:Cas9 RNP-complexes with the previously described target sequence located in exon 3 of *CCM3* [23]. Briefly, 6 µM sgRNA and 6 µM *S.p.* Cas9 protein (Integrated DNA Technologies, Coralville, IA, USA) were complexed in Opti-MEM I reduced serum medium (Thermo Fisher Scientific), and transfection complexes were formed in Opti-MEM with Lipofectamine Stem Transfection Reagent (Thermo Fisher Scientific). Cells were detached with StemPro Accutase (Thermo Fisher Scientific) and reverse transfected in Essential 8 medium (Thermo Fisher Scientific) supplemented with 10 µM ROCK inhibitor Y-27632 (Stemcell Technologies, Vancouver, Canada) on growth factor reduced Matrigel-coated 24-well plates (30 nM final RNP concentration; 150,000 cells and 2.0 µl Lipofectamine Stem Transfection Reagent). After 24 h, the medium was replaced with Essential 8 Flex medium without ROCK inhibitor. To establish clonal lines, genome-edited hiPSCs were seeded at a density of 0.5 cells/well on growth factor reduced Matrigel-coated 96-well plates and cultivated with Essential 8 Flex medium supplemented with CloneR (Stemcell Technologies). The genotypes of clonally expanded lines were determined by Sanger sequencing.

### Differentiation of hiPSCs to hBMEC-like cells

*CCM3*^+/+^ AICS-0054 hiPSCs, *CCM3*^+/+^ and *CCM3*^*−/−*^ AICS-0036 hiPSCS were differentiated to human brain microvascular endothelial-like cells (hBMEC-like cells) according to the protocol of Neal and colleagues [24] with minor modifications. Briefly, 158,000 cells were seeded in Essential 8 Flex medium containing 10 µM Y-27632 on growth factor reduced Matrigel-coated 6-well plates. After 24 h, differentiation was initiated by substituting Essential 8 Flex with Essential 6 medium (Thermo Fisher Scientific). The medium was changed daily for 4 days. On day 4, the medium was switched to Human Endothelial Serum-Free Medium (hESFM, Thermo Fisher Scientific) supplemented with FGF-2 (Miltenyi Biotec, Bergisch Gladbach, Germany), retinoic acid (Sigma-Aldrich, St. Louis, MO, USA), insulin (Sigma-Aldrich), holo-transferrin (Sigma-Aldrich), and sodium selenite (Sigma-Aldrich). After 48 h, cells were replated on 24-well plates coated with collagen IV from human placenta (Sigma-Aldrich) and human plasma fibronectin (Sigma-Aldrich). 24 h later, medium was changed to hESFM supplemented with insulin, holo-transferrin, and sodium selenite until confluency was reached. Differentiated hBMEC-like cells were passaged and expanded in EndoGRO-MV medium (Merck) supplemented with 1 ng/ml FGF-2. High expression of the tight junction proteins occludin and claudin-5 was verified by immunofluorescence microscopy. Cells were fixed with 4% PFA, stained for occludin (1:100, OC-3F10, Thermo Fisher Scientific) and claudin-5 (1:50, 4C3C2, Thermo Fisher Scientific) at 4 °C overnight, and incubated with a secondary antibody (1:200, A-11029, Thermo Fisher Scientific or 1:200, ab150114, abcam) for 2 h at room temperature. HBMEC-like cells were also seeded on matrigel-coated 96-wells at a density of 20.000 cells/well in hESFM supplemented with insulin, holo-transferrin, sodium selenite, and 50 ng/ml VEGF to verify tube formation. Images were acquired after 18 h. To study the proliferation characteristics of mutant hBMEC-like cells, *CCM3*^+/+^ AICS-0054 hBMEC-like cells and *CCM3*^+/+^ or *CCM3*^*−/−*^ AICS-0036 hBMEC-like cells were mixed in a 9:1 ratio (2500 cells per 96-well) and cultured for 6 days. On day 6, cells were fixed with 4% paraformaldehyde (PFA) or replated on 96-well plates (2500 cells per well). On day 12, the wells were fixed, and Hoechst 33342 (Thermo Fisher Scientific) staining was performed for subsequent fluorescent imaging analysis with the EVOS FL imaging system. The ratio of EGFP-tagged AICS-0036 cells compared to all cells was determined using the ImageJ software. Differentiated hBMEC-like cells were used in passages 3 and 5.

### Differentiation of hiPSCs to vascular organoids

hiPSCs were differentiated to vascular organoids according to the protocol of Wimmer and colleagues [25]. To study the proliferation characteristics of mutant cells, *CCM3*^+/+^ AICS-0054 hiPSCs and *CCM3*^+/+^ or *CCM3*^*−/−*^ AICS-0036 hiPSCS were mixed in a 9:1 ratio and differentiated to vascular organoids. Briefly, 800,000 hiPSCs were seeded in ultra-low attachment 6-well plates (Corning) in aggregation medium for 2 days until aggregates had formed. On day 0, mesodermal induction was initiated with CHIR99021 (Tocris Bioscience, Bristol, United Kingdom) and BMP-4 (Miltenyi Biotec) in N2B27 medium. Supplementation of VEGF-A (PeproTech, Inc., Rocky Hill, NJ, USA) and forskolin (Sigma-Aldrich) on day 3 induced vascular differentiation. On day 5, vascular aggregates were embedded in a collagen I-Matrigel matrix. VEGF-A, FGF-2 (Miltenyi Biotec), and FCS (Thermo Fisher Scientific) enriched StemPro-34 medium (Thermo Fisher Scientific) was added for vessel sprouting. Vascular network extraction was performed on day 10. On day 15, fully encapsulated organoids were fixed with 4% PFA, and nuclei staining was performed using Hoechst 33342 (Thermo Fisher Scientific). Fixed organoids were suspended in ibidi mounting medium (ibidi, Gräfelfing, Germany) and placed in 96-well cell imaging plates (Eppendorf AG, Hamburg, Germany). Imaging was performed using the Operetta CLS High-Content Imaging System (PerkinElmer, Waltham, MA, USA). Data were analyzed with the Harmony High-Content Imaging and Analysis Software (version 4.9, PerkinElmer).

Fusion organoids originating from the combination and co-culture of vascular networks acquired from *CCM3*^+/+^ and *CCM3*^−/−^ cells were used to study the effect of NSC59984 in hiPSC derived vascular organoids. 8 × 10^5^ AICS-0054 *CCM3*^+/+^ cells, AICS-0036 *CCM3*^+/+^ cells and AICS-0036 *CCM3*^−/−^ cells were differentiated into vascular networks as described previously [25]. Vascular networks derived from AICS-0054 *CCM3*^+/+^ hiPSCs were co-cultured with AICS-0036 *CCM3*^+/+^ or AICS-0036 *CCM3*^−/−^ vascular networks in the presence of 10 µM NSC59984. Medium supplemented with NSC59984 was changed every three days. On day 3 of co-culture, completely encapsulated fusion organoids were obtained and further cultivated until organoid examination on day 5 and day 10.

### Clonogenic and proliferation assays

Plating efficiencies were determined following a previously published protocol [26]. In brief, *CCM3*^+*/*+^ and *CCM3*^*−/−*^ CI-huVEC were seeded in 6-well plates as near-perfect single cell suspensions with either 100 or 250 cells per well. Colonies were fixed and stained after eight days with a glutaraldehyde solution (G6257, Sigma-Aldrich, final concentration 6%) and crystal violet (HT901, Sigma-Aldrich, final concentration 0.5%). The plating efficiency was defined as the ratio between the number of colonies and the number of plated cells. The proliferation rate of mutant and wild-type ECs in mono-culture was quantified with the CyQuant Cell Proliferation Assay Kit (C7026, Thermo Fisher Scientific). To discriminate the proliferation rates of *CCM3*^+*/*+^ and *CCM3*^*−/−*^ CI-huVEC in co-culture, the CyQuant Cell Proliferation Assay results were combined with mutant allele frequencies that had been determined by amplicon deep sequencing. In detail, measured RFU from the proliferation assay experiment were converted to cell numbers using a reference standard curve (RFU vs. cell number). These numbers represent the total cell number of a replicate at a given time point in the experiment. Subsequently, total cell number was multiplied by the allele frequency of a specific genotype. Allele frequencies were calculated from amplicon deep sequencing experiments performed in parallel with the proliferation assays. Since we used *CCM3*^*−/−*^ CI-huVEC in co-culture experiments that were either compound heterozygous or homozygous for loss-of-function alleles, the calculated allele frequency of a genotype corresponds to the proportion of cells with that genotype. For genotype-specific proliferation rates, the calculated cell number was normalized to the calculated cell number at day 0.

$$[{\mathrm{proliferation\,\, rate }\,\,(\mathrm{in \,\%})}_{\mathrm{genotype}}=\frac{{\left(\mathrm{RFU}*{\mathrm{F}}_{\mathrm{standard\,\,curve}}*{\mathrm{F}}_{\mathrm{ genotype\,\,allele\,\,frequency}}\right)}_{\mathrm{day\,\,d}}}{{\left(\mathrm{RFU}*{\mathrm{F}}_{\mathrm{standard\,\,curve}}*{\mathrm{F}}_{\mathrm{genotype\,\,allele\,\, frequency}}\right)}_{\mathrm{day}\,\,0}}*100\mathrm{\%}]$$

### Caspase-3, caspase-8, and caspase-9 activity assays

To analyze their sensitivity to apoptosis, mutant and wild-type ECs were seeded in 96-well plates with 15,000 cells per well and treated with staurosporine (Sigma-Aldrich) 24 h later. The Caspase-3 DEVD-R110 Fluorometric HTS Assay Kit (Biotium, Fremont, CA, USA), the Cell Meter Caspase 8 Activity Apoptosis Assay Kit (AAT Bioquest, Sunnyvale, CA, USA), and the Cell Meter Caspase 9 Activity Apoptosis Assay Kit (AAT Bioquest) were used following the manufacturer’s instructions.

### Protein extraction, antibody arrays, western blot and ELISA analyses

For the Human Apoptosis Antibody Array (ab134001, Abcam), proteins were extracted with PeqGold TriFast reagent (Peqlab-VWR, Radnor, PA, USA) and solubilized in buffer containing 8 M Urea, 2 M Thio-Urea, and 20 mM Tris. For the Human MAPK Phosphorylation Antibody Array (ab211061, Abcam), proteins were extracted according to the manufacturer's instructions. For Western Blot analyses, proteins were extracted with PeqGold TriFast reagent or RIPA Lysis and Extraction Buffer (Thermo Fisher Scientific). A Qubit Protein Assay Kit (Thermo Fisher Scientific) or a Micro BCA Protein Assay Kit (Thermo Fisher Scientific) was used to measure protein concentrations. The Human Apoptosis Antibody Array-Membrane Kit was used according to the manufacturer’s instructions to analyze the expression of apoptosis-related proteins in *CCM3*^+*/*+^ and *CCM3*^*−/−*^ CI-huVEC after 24 h of treatment with 0.05 µM staurosporine. The Human MAPK Phosphorylation Antibody Array Kit was used according to the manufacturer's instructions to analyze the expression of MAPK pathway markers in *CCM3*^+*/*+^ and *CCM3*^*−/−*^ CI-huVECs after co-culture. 600 µg of total protein was incubated for each membrane, and chemiluminescence signals were documented with a ChemiDoc XRS+ imager (Bio-Rad, Hercules, California, USA). Densitometry data were obtained using the ImageLab software (v6.0, Bio-Rad). For CCM3 and p21 Western Blot analyses, protein samples were suspended with Laemmli Sample Buffer (Bio-Rad) and heated at 95 °C for 5 min. For p53, heat denaturation was perfomed under reducing conditions. 20 µg (p21), 30 µg (CCM3) or 40 µg (p53) of total protein were separated on a 10% TGX Stain-Free FastCast SDS-polyacrylamide gel (Bio-Rad) and subsequently transferred to PVDF membranes. The iBind Flex Western System (Thermo Fisher Scientific) was used for immunostaining according to the manufacturer's instructions. The following primary antibodies were used: Anti-PDCD10/CCM3 (1:200, ab110531, Abcam or 1:200, ab180706, Abcam), Anti-p21 (1:1000, ab109520, Abcam), Anti-p53 (1:40, sc-126, Santa Cruz Biotechnology, Dallas, TX, USA) and Anti-GAPDH (1:500, PA1-16777, Thermo Fisher Scientific). A HRP-conjugated goat anti-rabbit immunoglobulin antibody (1:400, ab205718, Abcam) or a HRP-conjugated goat anti-mouse immunoglobulin antibody (1:400, ab205719, Abcam) with Precision Protein StrepTactin-HRP Conjugate (1:2000, Bio-Rad) were used as secondary antibodies. Detection of proteins was performed using Clarity Western ECL Substrate (Bio-Rad). Stain-free total protein and colorimetric protein bands were documented using a ChemiDoc XRS+ imager. The ImageLab software was used to calculate normalized band intensities. To calculate the relative protein expression, the volume intensities of the detected protein bands were normalized to the volume intensities of the corresponding GAPDH bands. The detection of GAPDH was performed after stripping the membrane with ROTI Free Stripping Buffer 2.2 plus (Carl Roth, Karlsruhe, Germany) for 1 h at room temperature.

To examine p53 activity, the p53 Transcription Factor Assay Kit (Cayman Chemical Company, Ann Arbor, MI, USA) was used according to manufacturer's instructions. Nuclear extracts were isolated from *CCM3*^+/+^ and *CCM3*^−/−^ cells using the Nuclear Extraction Kit (Cayman Chemical Company). 10 µg of nuclear extracts were incubated for each well at 4 °C overnight. Signal was measured at OD_450nm_ using an Infinite M200 Plate Reader (Tecan, Männedorf, Switzerland).

### Apoptosis compound library screen and amplicon deep sequencing

An apoptosis compound library with 189 small molecules was used in a high throughput screening approach [HY-L003(HY-LD-000001651); MedChem Express, Monmouth Junction, NJ, USA]. Co-cultures were treated with 10 µM of each substrate for 6 days. Compounds were added 5 h and 3 days after cell seeding. NSC59984 (IUPAC: (2E)-1-(4-Methyl-1-piperazinyl)-3-(5-nitro-2-furyl)-2-propen-1-one, HY19726, MedChem Express), isoalantolactone (IUPAC: (3aR,4aS,8aR,9aR)-8a-Methyl-3,5-bis(methylene)decahydronaphtho[2,3-b]furan-2(3H)-one, HY-N0780, MedChem Express), and GSK-872 (IUPAC: *N*-(6-propan-2-ylsulfonylquinolin-4-yl)-1,3-benzothiazol-5-amine, HY-101872, MedChem Express) were used for individual experiments with the indicated concentrations. DMSO-treatment (A994, Carl Roth) served as control. After 6 days, the fraction of mutant alleles in co-culture was analyzed by amplicon deep sequencing. In brief, the genomic target region was amplified by PCR. For NGS analysis, sequencing adapters and individual barcodes were introduced in a second PCR [22, 23]. Sequencing libraries were pooled and sequenced on a MiSeq instrument with 2 × 150 cycles (Illumina, San Diego, CA, USA). Data were analyzed with the SeqNext software (JSI Medical Systems, Ettenheim, Germany). The following compounds were tested for the ability to abrogate NSC59984-mediated inhibition of clonal expansion: UC2288 (Sigma-Aldrich), SCH772984 (Hycultec, Beutelsbach, Germany), and U0126-ETOH (Hycultec). Substances were supplemented 5 h and 3 days after seeding. Amplicon deep sequencing was perfomed at day 6.

### Integrin antibody-array, ITGB4 qPCR and staining, FACS sorting

The β-Integrin-mediated Cell Adhesion Array Kit (ECM534, Sigma-Aldrich) was used according to the manufacturer’s instructions to quantify cell surface expression of *β*_1_, *β*_2_, *β*_3_, *β*_4_, *α*_V_*β*_5_, and *α*_5_*β*_1_ integrins of *CCM3*^+*/*+^ and *CCM3*^*−/−*^ CI-huVECs. Fluorescence intensity was measured with the Qubit 4 Fluorometer (Thermo Fisher Scientific). For immunofluorescent imaging, CI-huVECS were cultured on 96-well plates until confluency was reached. Mouse anti-integrin *β*4 (ITGB4) antibody (1:100, MAB2059Z, Merck, Darmstadt, Germany) was added to the culture medium, and cells were incubated for 1 h at 37 °C and 5% CO_2_. Cells were washed three times with culture medium and incubated for 1 h at 37 °C and 5% CO_2_ with goat anti-mouse, Alexa Fluor 488 secondary antibody (1:200, A-11029, Thermo Fisher Scientific). Subsequently, cells were washed three times in culture medium and imaged with the EVOS FL imaging system (Thermo Fisher Scientific). For qPCR analysis, mRNA was transcribed into cDNA using the First Strand cDNA Synthesis Kit (Thermo Fisher Scientific). SYBR Green-based qPCR analysis was performed on a Roche Light Cycler 480 instrument (Roche, Mannheim, Germany) to validate deregulated gene expression of *ITGB4*. The gene *RPLP0* served as an endogenous control. The following primer pairs purchased from Integrated DNA Technologies were used: *ITGB4—*5′-CTACTACGAGAAGCTTCACAC-3′ and 5′-GACCCAGTCCTCGTCTTCTG-3′; *RPLP0—*5′-TCGACAATGGCAGCATCTAC-3′ and 5′-ATCCGTCTCCACAGACAAGG-3′. For FACS sorting of co-cultured CI-huVECs, 1–2 million cells were seeded on T75-flasks in a 2:3 ratio of mutant to wild-type cells and cultured for three days. Cells were dissociated with Accutase and stained with mouse anti-integrin *β*4 antibody (1:200) and goat anti-mouse, Alexa Fluor 488 secondary antibody (1:200). Cells were resuspended in PBS and flow cytometry was used for sorting (BD FACSAria III Cell Sorter; BD Biosciences, Franklin Lakes, NJ, USA). The RNA of ITGB4^high^ and ITGB4^low^ fractions were extracted in TRI Reagent for fluid samples (Sigma-Aldrich). The purity of fractions was confirmed on DNA, RNA, and protein level. DNA and RNA were extracted to evaluate purity via amplicon deep sequencing and qPCR, respectively. For transcript analyses, the *CCM3* qPCR assay PrimeTime Hs.PT.58.38574999 (Integrated DNA Technologies) was used.

### RNA sequencing and qPCR analyses

Extracted RNA was purified using the Direct-zol RNA MiniPrep Plus Kit (Zymo Research, Irvine, CA, USA). If necessary, isolated RNA was concentrated with the RNA Clean & Concentrator-5 Kit (Zymo Research). A Qubit 4.0 (Thermo Fisher Scientific) and the Qubit RNA BR Assay Kit (Thermo Fisher Scientific) were used to measure RNA concentrations. RNA sample integrity was controlled on a 2100 Bioanalyzer using the RNA 6000 Nano Kit (Agilent, Santa Clara, CA, USA). RNA-seq libraries were prepared via polyA selection and sequenced on an Illumina NovaSeq platform (Illumina) with 2 × 150 cycles by GENEWIZ (Leipzig, Germany). Reads were trimmed to remove adapter sequences using Trimmomatic v.0.36 and mapped to the Homo sapiens GRCh37 genome with STAR aligner v.2.5.2b. The software featureCounts from the Subread package v.1.5.2 was used to extract unique gene hit counts. For targeted RNA-analysis, a QIAseq Targeted Human Apoptosis and Cell Death RNA Panel (RHS-002Z, QIAGEN, Hilden, Germany) was used to prepare RNA sequencing libraries according to the manufacturer’s instructions. After quality control with the High Sensitivity DNA Kit on a 2100 Bioanalyzer instrument (Agilent), the pooled libraries were sequenced on a MiSeq (Illumina) with 1 × 151 cycles. Differential gene expression of RNA sequencing data was carried out using the QIAseq Targeted RNA Primary and Secondary Analysis Tool (QIAGEN). The PrimeTime assays Hs.PT.58.40874346.g and Hs.PT.58.38974274 (Integrated DNA Technologies) were used to measure *CDKN1A* and *GADD45A* gene expression. The gene *RPLP0* served as an endogenous control.

### Statistical analysis

The GraphPad Prism software (v.8.0.1, GraphPad Software, LA Jolla, CA, USA) was used for statistical analysis. Unless stated otherwise, all data are presented as mean and standard deviation (SD). For analyses of two or more groups, multiple *t* tests, one- and two-tailed Student’s *t* test, and one- and two-way ANOVA were used. Dunnett’s or Šidák's corrections were applied for multiple comparisons. *P* values or adjusted *p* values < 0.05 were regarded as statistically significant. For genome-wide RNA-seq, differential gene expression analysis was performed using DESeq2. *P* values and log2 fold changes were generated using the Wald test. Genes were regarded as differentially expressed between compared groups with an adjusted *p* value < 0.05 and an absolute log2 fold change > 1. Gene ontology analysis was performed using the GeneSCF v.1.1-p2 software combined with the goa_human GO list to cluster genes based on biological processes.

## Results

### *CCM3* inactivation alone does not trigger EC proliferation

**Fig. 1:**
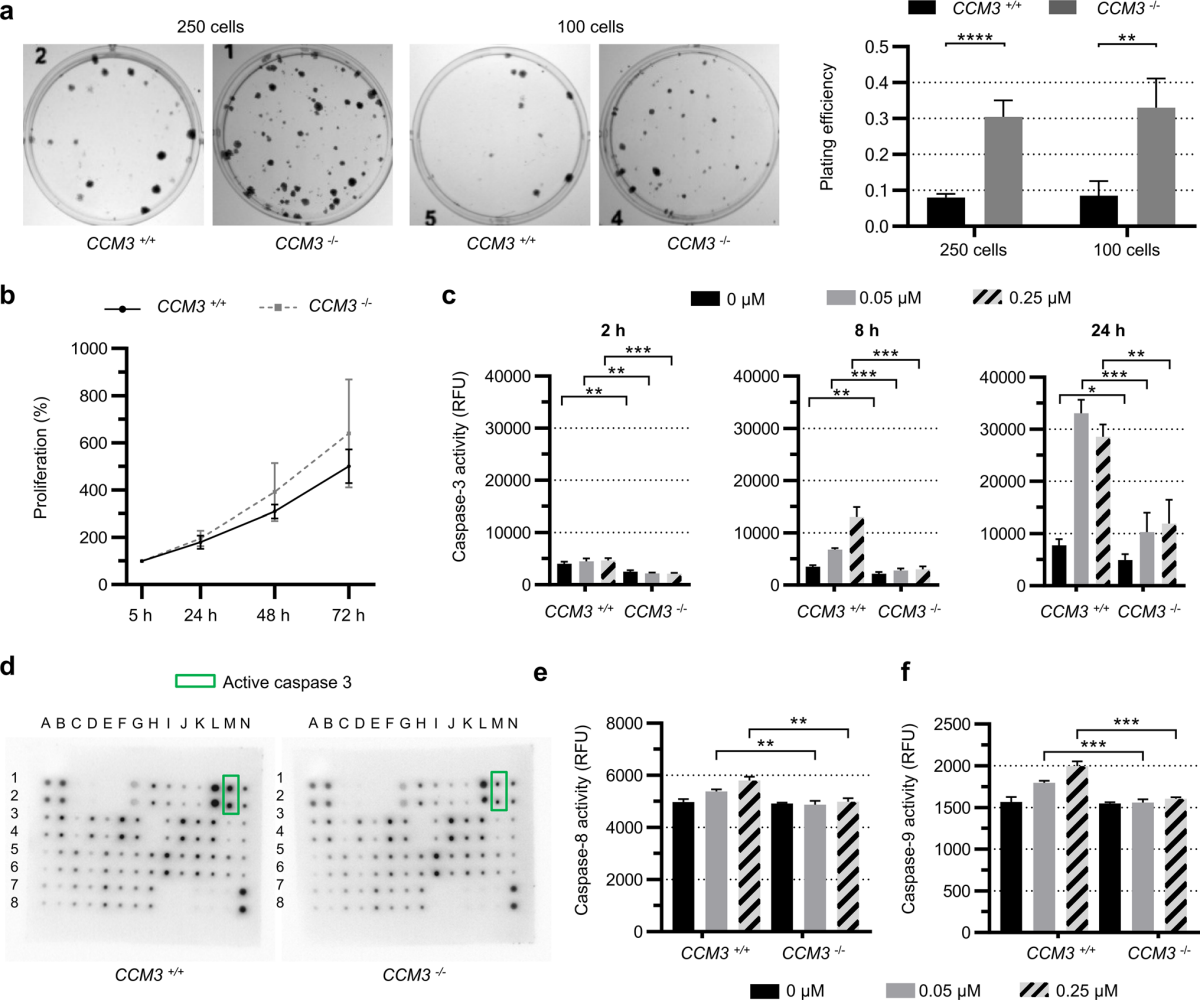
Inactivation of *CCM3* gene expression in human ECs causes resistance to apoptosis and increased clonogenicity. **a** *CCM3*^*−/−*^ and *CCM3*^+*/*+^ CI-huVECs were seeded as (near-perfect) single-cell suspensions in 6-well plates with either 250 or 100 cells per well. Colonies were stained with crystal violet after eight days. Representative images are shown for both genotypes. The plating efficiency of *CCM3*^*−/−*^ CI-huVECs was significantly increased under both seeding conditions (*n* = 4 per genotype). **b** *CCM3* inactivation did not significantly enhance proliferation of CI-huVECs under standard culture conditions (*n* = 9 per genotype). **c** CI-huVECs were treated with staurosporine (0.05 µM or 0.25 µM) to induce apoptotic cell death. After 2, 8, and 24 h, the caspase-3 activity was markedly reduced in *CCM3*^*−/−*^ CI-huVECs (*n* = 3 per genotype). **d** A human apoptosis antibody array assay verified the reduction of active caspase-3 levels in staurosporine-treated (0.05 µM, 24 h) *CCM3*^*−/−*^ CI-huVECs (*n* = 3 per genotype). Representative array membranes are shown for both genotypes. Green rectangles mark active caspase-3. No significant differences were found for other apoptosis markers. **e**, **f** The activities of caspase-8 (**e**) and caspase-9 (**f**) were also slightly reduced in staurosporine-treated (0.05 µM or 0.25 µM, 8 h) *CCM3*^*−/−*^ CI-huVECs (*n* = 3 per genotype). RFU = relative fluorescence units. Data are presented as mean and SD. Student’s two-tailed *t* tests (**a–c**, **e**, **f**) were used for statistical analyses: *P < 0.05, **P < 0.01, ***P < 0.001, ****P < 0.0001

To compare the growth and survival characteristics of wild-type and mutant ECs, we first plated single-cell suspensions of *CCM3*^+*/*+^ and *CCM3*^*−/−*^ CI-huVECs, and analyzed their ability to grow into colonies. We observed a significantly higher clonogenic capacity of mutant CI-huVECs. The plating efficiency of mutant compared to wild-type ECs was actually three times higher (Fig. 1a). However, CI-huVECs in which the *CCM3* gene had been disrupted by CRISPR/Cas9 genome editing did not display significantly different proliferation rates (Fig. 1b). Next, we stimulated apoptotic cell death in *CCM3*^*−/−*^ and *CCM3*^+*/*+^ CI-huVECs with staurosporine and found significantly higher survival rates of mutant ECs (Online Resource 2). Two, eight, and twenty-four hours after staurosporine treatment, the activity of caspase-3, which is a major effector caspase in apoptosis, was also significantly lower in *CCM3*^*−/−*^ CI-huVECs (Fig. 1c). Profiling the relative expression of 43 apoptosis-related proteins verified the reduced fraction of active caspase-3 but revealed no other significantly up- or downregulated targets in *CCM3*^*−/−*^ CI-huVECs (Fig. 1d, Online Resource 3). Caspase-3 is proteolytically activated by upstream caspases of the intrinsic and extrinsic pathways of apoptosis. Our protein array did not specifically detect the active forms of caspase-8 (extrinsic pathway) and caspase-9 (intrinsic pathway). Therefore, we used specific fluorogenic indicators to measure their activities. Both were slightly reduced in mutant compared to wild-type ECs after staurosporine treatment (Fig. 1e, f). These results demonstrate that mutant ECs have a survival advantage under specific stress conditions. However, unstressed *CCM3*^*−/−*^ mono-cultures do not show abnormal proliferation.

### Only direct co-culture with wild-type cells stimulates abnormal proliferation of mutant ECs

**Fig. 2:**
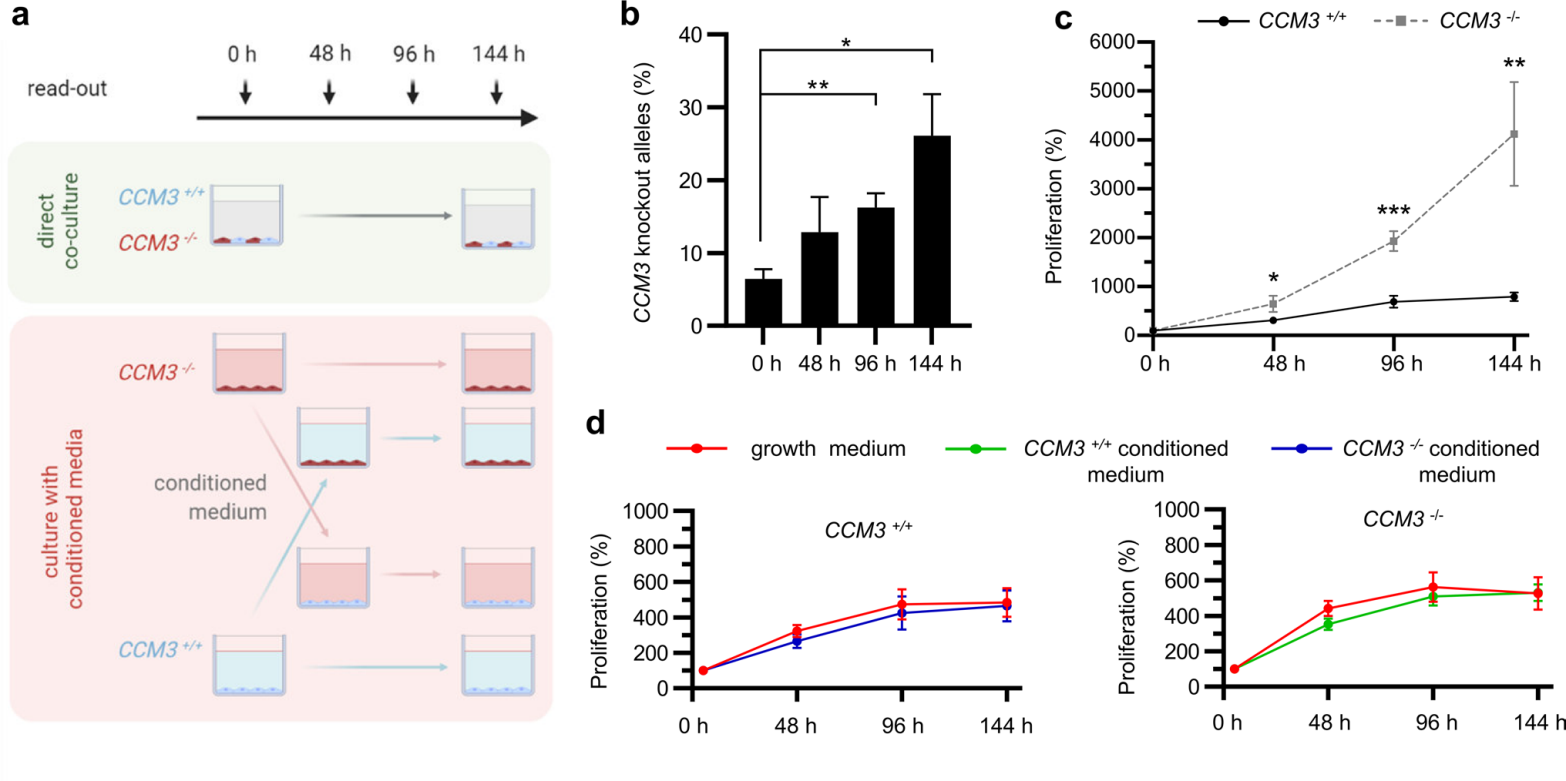
*CCM3*^*−/−*^ CI-huVECs demonstrate increased proliferative activity when directly co-cultured with *CCM3*^+*/*+^ ECs. **a** *CCM3*^*−/−*^ CI-huVECs were either directly co-cultured with *CCM3*^+*/*+^ CI-huVECs or with conditioned medium of *CCM3*^+*/*+^ CI-huVECs. In parallel, *CCM3*^+*/*+^ CI-huVECs were cultured with conditioned medium of *CCM3*^*−/−*^ CI-huVECs. Knockout and wild-type CI-huVECs cultured in unconditioned growth medium served as controls. Proliferation was analyzed at the indicated time points. **b**, **c** In direct co-culture, the proportion of *CCM3* knockout alleles markedly increased over time (**b**), and *CCM3*^*−/−*^ CI-huVECs proliferated significantly more (**c**) (*n* = 3 per condition). **d** *CCM3*^*−/−*^ CI-huVECs were cultured with conditioned medium from *CCM3*^+*/*+^ CI-huVECs and vice versa. Unconditioned growth medium was used as control. The conditioned medium alone did not influence the proliferation rate of *CCM3*^*−/−*^ CI-huVECs (*n* = 3 per condition). Data are presented as mean and SD. Student’s two-tailed *t* tests were used for statistical analyses: *P < 0.05, **P < 0.01, ***P < 0.001

To mimic the mosaic endothelial pattern in CCM tissue, in which the number of mutant ECs is usually much lower than the number of wild-type cells [15–18], we co-cultured *CCM3*^*−/−*^ with *CCM3*^+*/*+^ CI-huVECs in a 1:9 ratio for six days (Fig. 2a). When we combined the results of fluorescence-based cell proliferation assays with amplicon deep sequencing data, we found increasing mutant allele frequencies over time (Fig. 2b) and significantly higher proliferation rates of mutant ECs compared to wild-type cells in co-culture (4124% vs. 793% after 144 h, Fig. 2c). This effect was also evident in co-cultures with very low mutant:wild-type ratios (from 5:95 to 1:99, Online Resource 4). However, it completely vanished upon reversal of this ratio (from 95:5 to 99:1, Online Resource 4). Next, we asked whether the proliferative advantage of mutant ECs in co-culture depends on cell–cell interactions or can also be triggered by paracrine signaling alone. Thus, we studied the effect of conditioned medium on the proliferation rates of *CCM3*^*−/−*^ and *CCM3*^+*/*+^ CI-huVECs (Fig. 2a). When we cultured mutant ECs in conditioned medium of wild-type ECs, we detected no increased proliferation. In line with this observation, the conditioned medium of mutant ECs did not affect the mitotic activity of wild-type cells (Fig. 2d). Thus, direct cell contact with *CCM3*^+*/*+^ CI-huVECs is indispensable to trigger the proliferation of *CCM3*^*−/−*^ ECs in co-culture.

### CCM3 acts as suppressor of abnormal proliferation in vascular ECs but not in hiPSCs

**Fig. 3:**
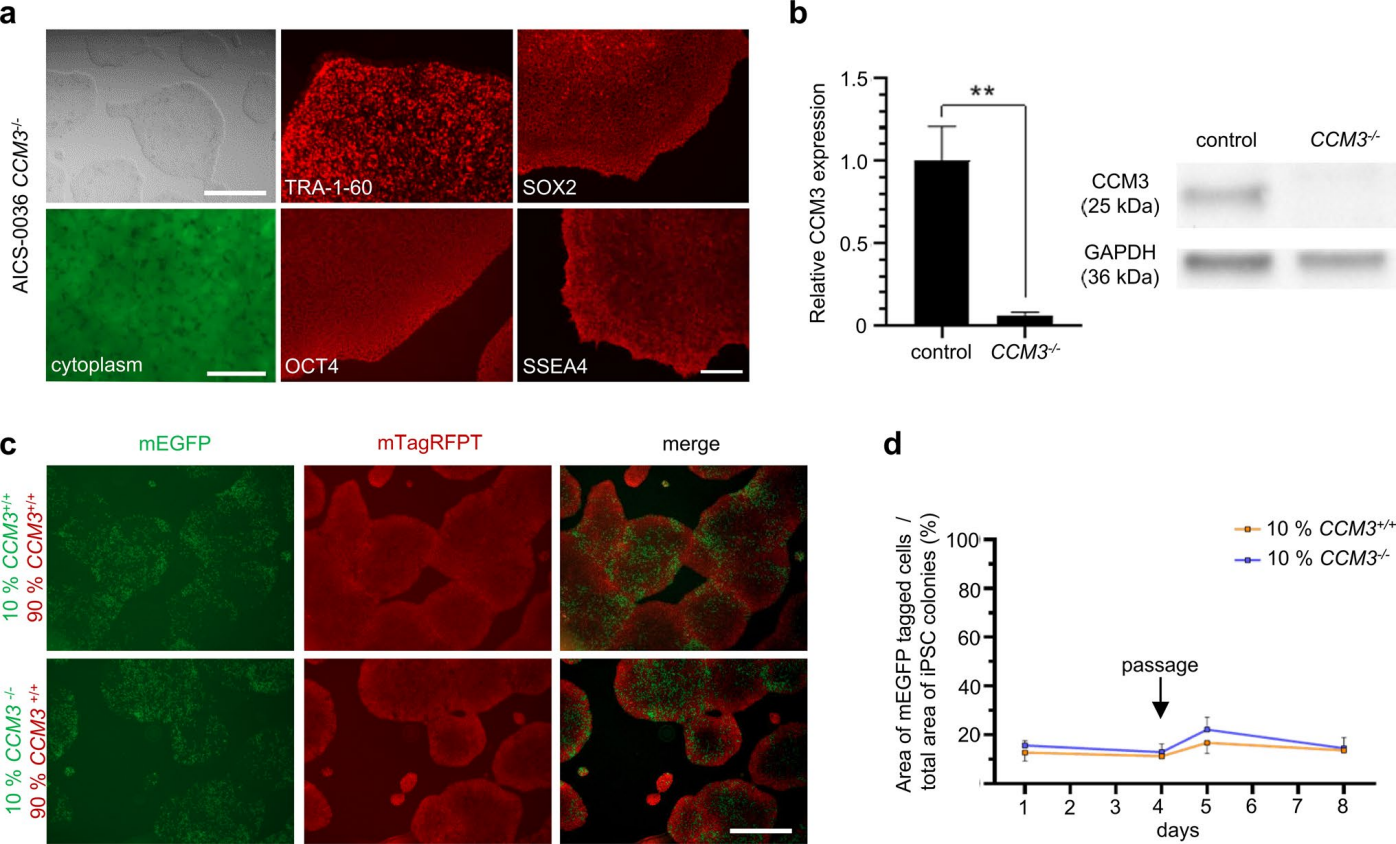
CCM3-deficient hiPSCs show no abnormal proliferation in co-culture with wild-type hiPSCs. **a** *CCM3*^−/−^ AICS-0036 hiPSC demonstrated typical hiPSC morphology (representative images; top left, scale bar: 500 µm) and strong expression of the pluripotency markers TRA-1–60, OCT4, SOX2, and SSEA4 (representative images; middle and right panels, scale bar: 200 µm). The mEGFP-tagged cytosol of *CCM3*^−/−^ AICS-0036 hiPSC is shown in the bottom left subpanel (scale bar: 50 µm). **b** Western blot analysis verified CCM3 inactivation in *CCM3*^−/−^ AICS-0036 hiPSCs. GAPDH was used as a loading control (*n* = 3 per genotype). **c**, **d** *CCM3*^−/−^ AICS-0036 hiPSCs were mixed with *CCM3*^+/+^ AICS-0054 hiPSCs in a 1:9 ratio and co-cultured for 8 days. *CCM3*^+/+^ AICS-0036 mixed with *CCM3*^+/+^ AICS-0054 hiPSCs served as controls (*n* = 3 per genotype). Representative images from day eight are shown in c (scale bar: 500 µm). Data are presented as mean and SD. Student’s two-tailed *t* tests (**b**, **d**) were used for statistical analyses: **P < 0.01

**Fig. 4:**
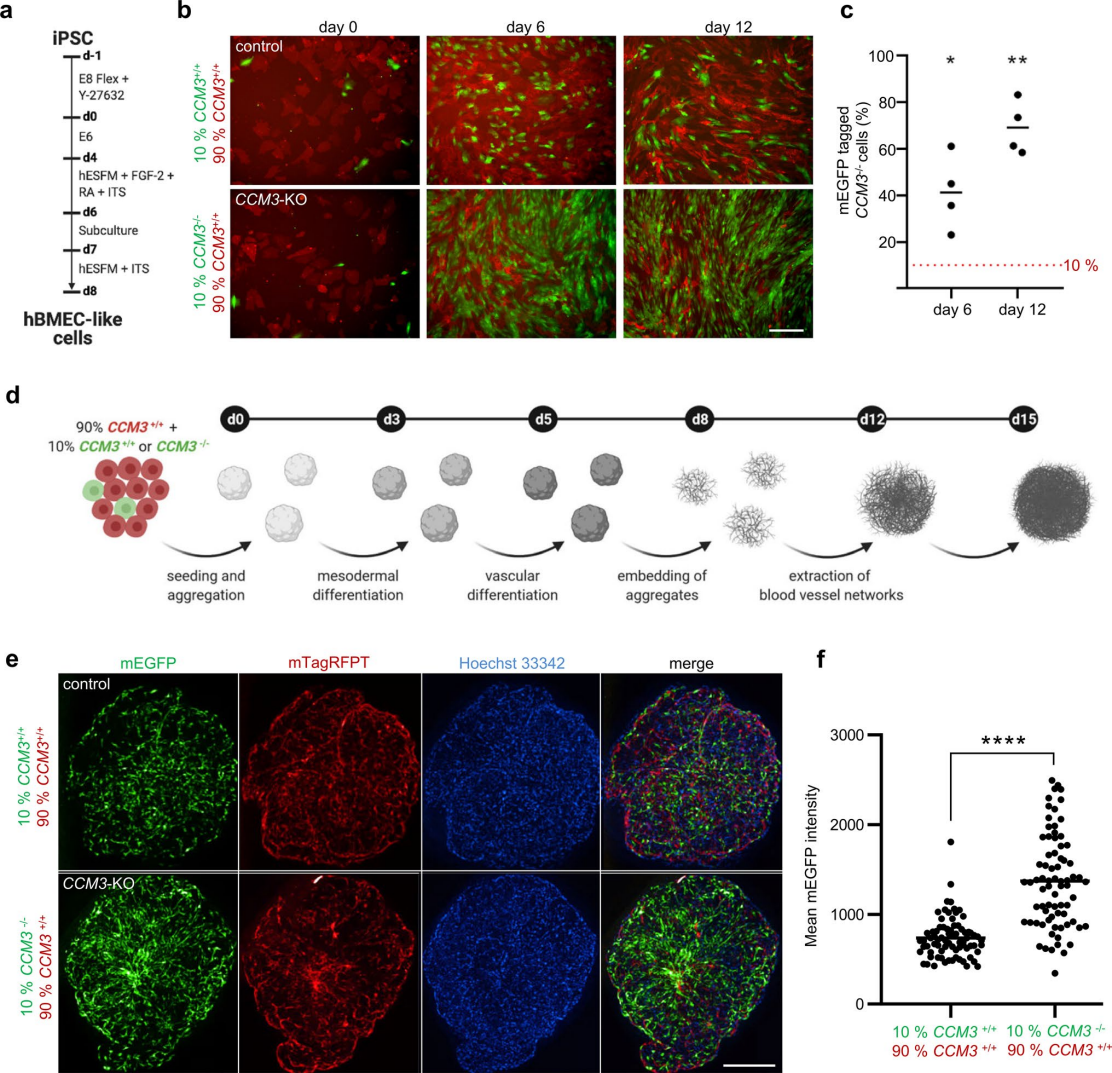
Human iPSC-derived *CCM3*^*−/−*^ ECs exhibit a tumor-like proliferative behavior in co-culture with wild-type ECs and in mosaic vascular organoids. **a–c** Brain microvascular endothelial-like cells (hBMEC-like cells) were differentiated from *CCM3*^−/−^ AICS-0036 (mEGFP), *CCM3*^+/+^ AICS-0036 (mEGFP), and *CCM3*^+/+^ AICS-0054 (mTagRFPT) hiPCS (**a**) and mixed in a 1:9 ratio (**b**, **c**). *CCM3*^−/−^ hBMEC-like cells (green) demonstrated an abnormal proliferation in co-culture with *CCM3*^+/+^ hBMEC-like cells (*n* = 4 per genotype; b, scale bar: 200 µm). **d** *CCM3*^−/−^ AICS-0036 (mEGFP) and *CCM3*^+/+^ AICS-0054 (mTagRFPT) hiPCS were mixed in a 1:9 ratio and differentiated to vascular organoids. *CCM3*^+/+^ AICS-0036 mixed with *CCM3*^+/+^ AICS-0054 hiPSCs served as controls. **e** Abnormal proliferation of *CCM3*^−/−^ cells was observed in mosaic vascular organoids (lower left panel, scale bar: 300 µm). **f** Statistical analyses from 77 KO/WT to 75 WT/WT mosaic organoids differentiated in three independent biological replicates. Shown is the mean mEGFP fluorescence intensity. Data are presented as mean and individual data points. One sample *t* test (**c**) or Student’s two-tailed *t* test (**f**) was used for statistical analyses: *P < 0.05, **P < 0.01, ****P < 0.0001

We next asked whether *CCM3* gene disruption also triggers abnormal proliferation of mutant hiPSCs in co-culture with wild-type hiPSCs. To answer this question, we used CRISPR/Cas9 genome editing to inactivate *CCM3* gene expression in mEGFP-tagged AICS-0036 hiPSCs (Online Resource 5). The CCM3 knockout had neither an effect on the morphology of the hiPSC colonies nor on the expression of the pluripotency markers TRA-1-60, SOX2, OCT4, and SSEA4 (Fig. 3a). After we had excluded recurrent genomic instabilities and verified the CCM3 knockout on protein level (Fig. 3b, Online Resource 5), mEGFP-tagged *CCM3*^*−/−*^ AICS-0036 hiPSCs and mTagRFPT-tagged *CCM3*^+*/*+^ AICS-0054 hiPSCs were mixed in a 1:9 ratio and cultured for up to eight days. Interestingly, no abnormal proliferation of mutant hiPSCs was observed in co-culture with wild-type hiPSCs (Fig. 3c, d). To exclude a cell-type-specific effect of the striking cancer-like proliferation of *CCM3*^*−/−*^ CI-huVECs in co-culture, we differentiated hiPSCs into human brain microvascular endothelial-like cells (hBMEC-like cells; Fig. 4a, Online Resource 6) and co-cultured *CCM3*^*−/−*^ and *CCM3*^+*/*+^ hBMEC-like cells in a 1:9 ratio. Within 6 to 12 days, the percentage of mutant cells in co-culture increased to 41.2% (SD 15.97) and 69.0% (SD 11.42), respectively (Fig. 4b, c). No abnormal proliferation was observed in co-cultures of mEGFP-tagged *CCM3*^+*/*+^ hBMEC-like cells and mTagRFPT-tagged *CCM3*^+*/*+^ hBMEC-like cells (Fig. 4b).

In vivo, endothelial proliferation acts in concert with differentiation, migration, and interaction of ECs with other cell types and the extracellular matrix [27]. This complex interplay can hardly be reproduced in 2D cultures alone. Thus, we utilized hiPSC-derived vascular organoids which have been described as powerful tool to study vasculopathies in 3D cultures [28]. When we differentiated co-cultures of mutant and wild-type hiPSCs to mosaic vascular organoids (Fig. 4d), we found no apparent defects of the vascular network but a strikingly increased proliferation of mutant cells (Fig. 4e, f). Like in human CCM lesions, CCM3-deficient cells did not form clusters but were found in a mosaic pattern with wild-type cells instead. Together, these results prompted us to address the abnormal mutant cell proliferation and their interaction with wild-type cells in more detail.

### Co-culture activates cytokine signaling in wild-type and cell proliferation pathways in mutant ECs

**Fig. 5:**
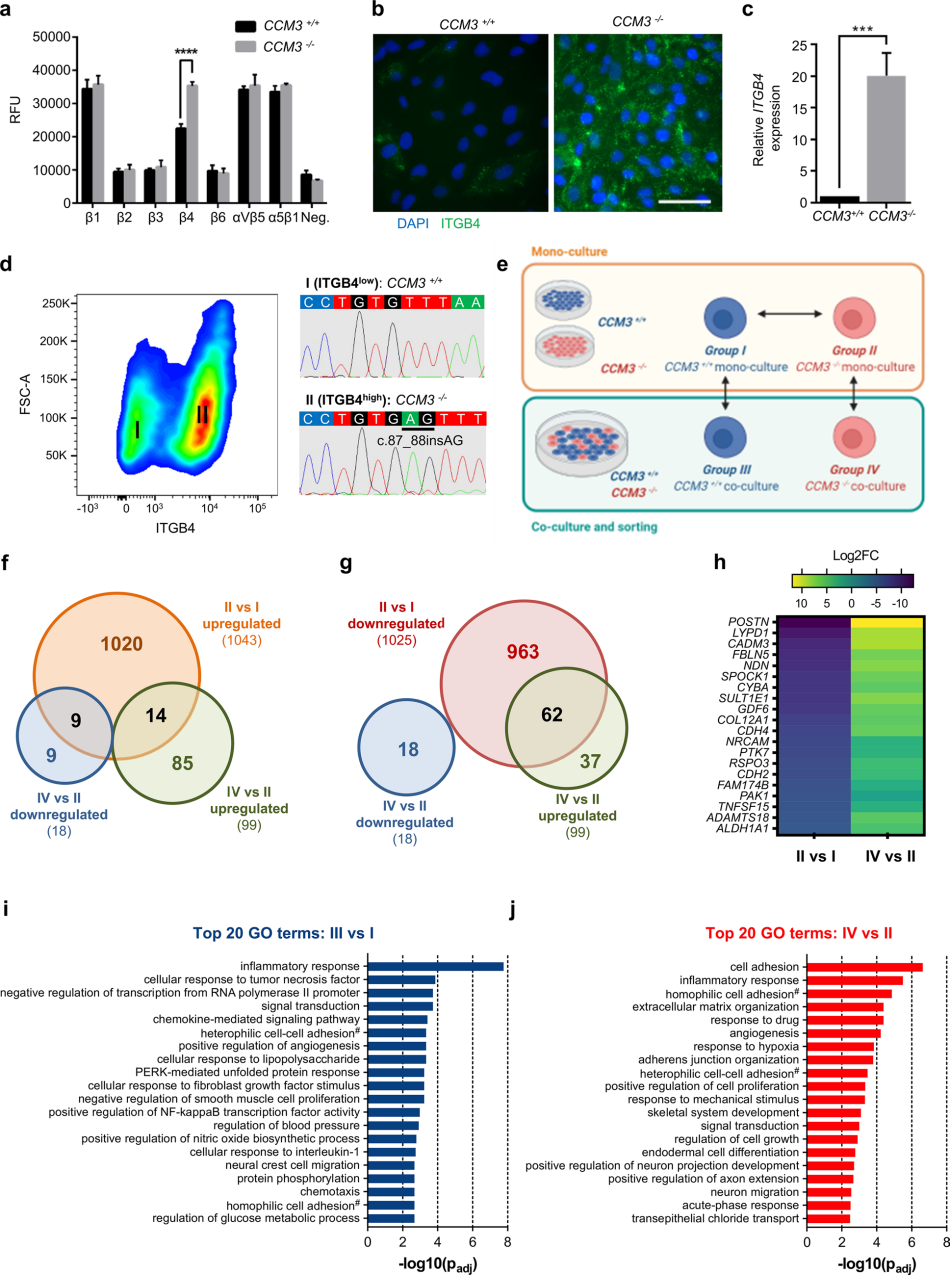
Chemokine signaling in wild-type ECs becomes deregulated in co-culture with *CCM3*^*−/−*^ ECs. **a** A β-integrin-mediated cell adhesion array was used to analyse the surface expression of different integrin β subunits on *CCM3*^*−/−*^ CI-huVECs (*n* = 3 per group). **b**, **c** Immunofluorescence imaging and RT-qPCR analysis verified upregulation of *ITGB4* gene expression in *CCM3*^*−/−*^ CI-huVECs (scale bar: 100 µm). Data are presented as mean and SD (*n* = 3 per group). **d** Using high integrin beta 4 expression as marker for *CCM3*^*−/−*^ CI-huVECs, mutant ECs could be efficiently sorted from mutant/wild-type co-cultures by fluorescence activated cell sorting. Sanger sequencing of sorted ITGB4^low^ (I) and ITGB4^high^ (II) cell populations verified high purity of *CCM3*^+*/*+^ and *CCM3*^*−/−*^ CI-huVECs, respectively. **e** *CCM3*^+*/*+^ and *CCM3*^*−/−*^ CI-huVECs were co-cultured, sorted by FACS and analyzed by RNA sequencing. *CCM3*^+*/*+^ and *CCM3*^*−/−*^ CI-huVEC mono-cultures served as controls. **f**, **g** Venn diagrams illustrate the overlap of genes significantly up- or downregulated in *CCM3*^*−/−*^ CI-huVEC mono-cultures (group II vs group I) and genes that are significantly up- or downregulated in *CCM3*^*−/−*^ CI-huVECs by co-culture with *CCM3*^+*/*+^ CI-huVECs (group IV vs group II). **h** Shown is a heatmap with the 20 most downregulated genes in *CCM3*^*−/−*^ CI-huVEC mono-cultures (left column; group II vs group I). The right column illustrates how co-culture with wild-type ECs affects the expression of these genes in *CCM3*^*−/−*^ CI-huVECs (group IV vs group II). **i**, **j** Significantly up- or downregulated genes were also subjected to gene ontology analysis. Shown are the top 20 of significantly enriched biological process GO terms (*n* = 3 per group for **e**–**j**). ^#^via plasma membrane cell adhesion molecules. Multiple *t* tests (**a**), Student’s two-tailed *t* test (**c**), and Fisher exact test (**i**, **j**) were used for statistical analyses: ***P < 0.001, ****P < 0.0001.* p*_*adj*_ = adjusted *p* value

A better understanding of the interplay between mutant ECs and wild-type or heterozygous ECs in mosaic CCM lesions appears to be essential for the development of targeted therapeutic approaches. Therefore, we decided to study the gene expression profiles of *CCM3*^*−/−*^ and *CCM3*^+*/*+^ CI-huVECs in mono-culture and direct co-cultures. Using a ß-integrin-mediated cell adhesion array and specific immunofluorescence imaging, we identified high expression of the integrin subunit *ß*4 (ITGB4), which is known to form a heterodimer with integrin α6, as a marker for *CCM3*^*−/−*^ CI-huVECs (Fig. 5a, b). Consistent with published gene expression data of other groups (Online Resource 7), *ITGB4* mRNA levels were upregulated 20-fold in *CCM3*^*−/−*^ CI-huVECs (Fig. 5c). Integrin *α*6*β*4 is a specific receptor for laminin-332 and the ability of mutant ECs to bind exogenous laminin-332 was consequently increased (Online Resource 8). However, exogenous laminin-332 did not trigger the proliferation of *CCM3*^*−/−*^ CI-huVECs (data not shown).

FACS sorting of co-cultured *CCM3*^*−/−*^ and *CCM3*^+*/*+^ CI-huVECs for ITGB4^high^ and ITGB4^low^ cells resulted in two distinct populations (Fig. 5d). Amplicon deep sequencing, qPCR and western blot analyses verified high purities of sorted wild-type and mutant ECs, respectively (Online Resource 8). Based on these results, we set up a genome-wide gene expression profiling experiment (Fig. 5e). Focussing on *CCM3*^*−/−*^ and *CCM3*^+*/*+^ CI-huVECs in mono-culture*,* 1043 genes were found to be significantly upregulated, and 1025 were downregulated upon *CCM3* gene disruption (Fig. 5f, g, Online Resource 9). Interestingly, *CCM3*^*−/−*^ CI-huVECs in direct co-culture with *CCM3*^+*/*+^ CI-huVECs upregulated 99 genes and downregulated another 18 genes (Fig. 5f, g). A GO term enrichment analysis revealed deregulation of cell adhesion and proliferation pathways in *CCM3*^*−/−*^ CI-huVECs under co-culture conditions (Fig. 5j). It was surprising to notice that among the upregulated genes in *CCM3*^*−/−*^ CI-huVECs under co-culture conditions were especially those that were highly downregulated upon *CCM3* gene disruption (Fig. 5g, h). For example, *POSTN* gene expression, which was hardly detectable in *CCM3*^*−/−*^ CI-huVECs under mono-culture conditions, was upregulated to near-normal levels in co-culture (Fig. 5h). Once in contact with wild-type ECs, mutant ECs also upregulated the expression of genes coding for other extracellular matrix components, e.g., *FBLN5* (fibulin-5) and *COL12A1* (collagen type XII alpha 1 chain). Furthermore, genes coding for cadherin superfamily members, e.g., *CDH2* (N-cadherin), *CDH4* (R-cadherin), *CDH11* (OB-cadherin), and *PCDH7* (protocadherin-7), were significantly upregulated (Online Resource 9). In *CCM3*^+*/*+^ CI-huVECs, on the other hand, chemokine/cytokine-related pathways were found to be deregulated by co-culture with *CCM3*^*−/−*^ CI-huVECs (Fig. 5i). In particular, wild-type ECs upregulated various chemokine genes, e.g., *CCL2* (MCP-1), *CX3CL1* (fractalkine), *CCL7* (MCP-3), *CXCL2* (MIP-2a/GRO2), *CXCL3* (MIP-2b/GRO3), and *CXCL8* (IL-8), but downregulated *POSTN* and *CDH11* in co-culture with mutant ECs (Online Resource 9). Taken together, these observations prove that mutant and wild-type ECs interact with each other and suggest that CC and CXC chemokines play a role in this complex regulatory interplay.

### Compound screening identifies NSC59984 as candidate drug to block abnormal proliferation of mutant ECs

**Fig. 6:**
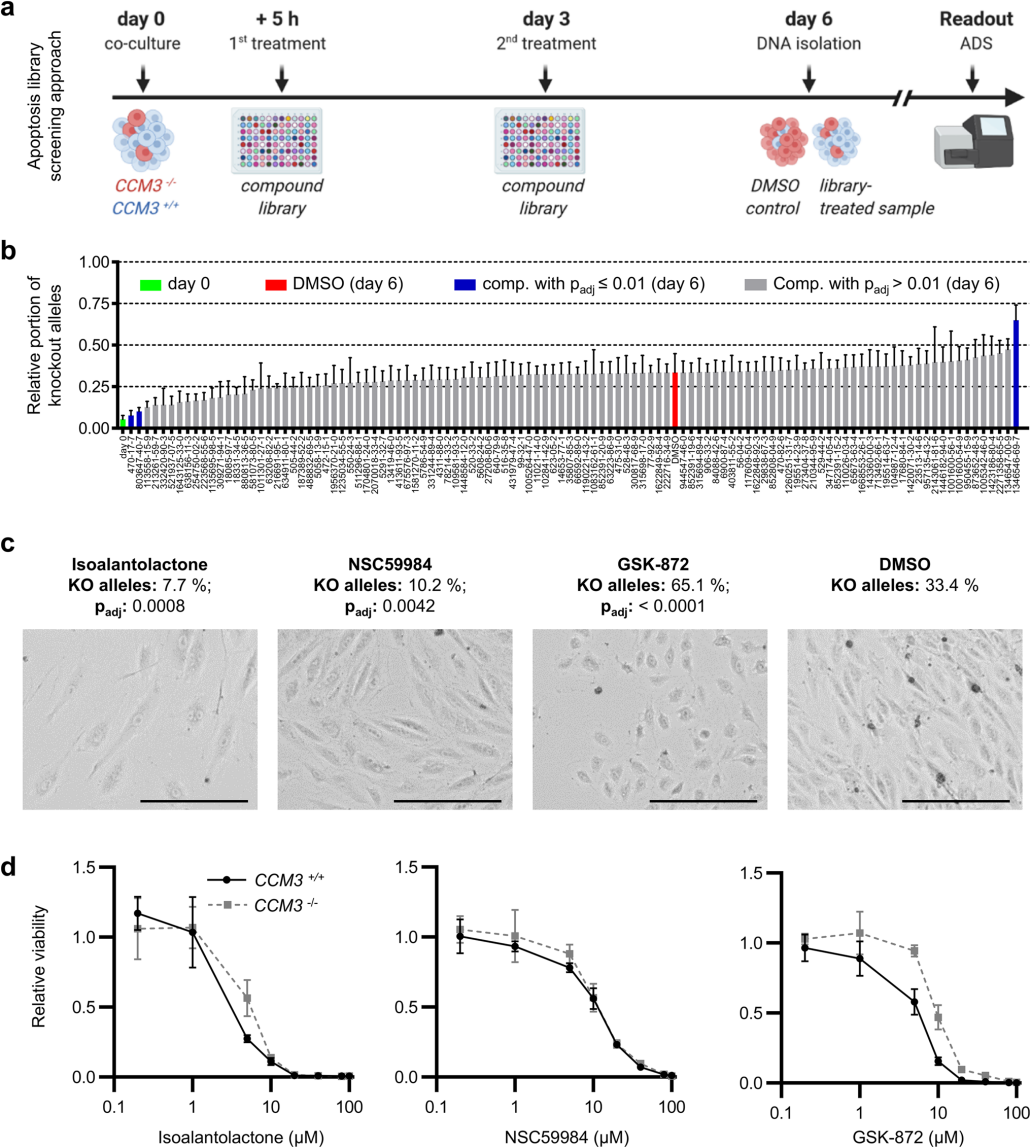
NSC59984 efficiently blocks the abnormal proliferation of *CCM3*^*−/−*^ CI-huVECs in co-culture. **a** Depicted is a scheme of the apoptosis library screening approach. Co-cultures of *CCM3*^*−/−*^ and *CCM3*^+*/*+^ CI-huVECs were treated with 10 µM of each library compound on day 0 and day 3. DNA samples collected at day 6 were used to determine frequencies of *CCM3* knockout alleles with amplicon deep sequencing (ADS). DMSO treatment served as control. **b** NSC59984 and isoalantolactone reduced the portion of *CCM3* knockout alleles in co-culture. In contrast, GSK-872 further enhanced the abnormal proliferation of *CCM3*^*−/−*^ CI-huVECs (*n* = 4 per group). **c** Bright-field microscopy demonstrated that cells treated with NSC59984 had a normal EC morphology while isoalantolactone treatment induced severe morphological changes and significant cell death (10 µM of each compound; scale bar: 200 µm). After GSK-872 treatment, cells had a compact morphology that is a known feature of *CCM3*^*−/−*^ CI-huVECs (*n* = 3 per group). **d** No cell viability differences were observed between *CCM3*^+*/*+^ and *CCM3*^*−/−*^ CI-huVECs 72 h after NSC59984 or isoalantolacone treatment, whereas *CCM3*^+*/*+^ CI-huVECs were more sensitive to GSK-872 treatment than mutant ECs (*n* = 3 per group). *Comp.* = library compound. *p*_*ad*j_ = adjusted *p* value. Data are presented as mean and SD. One-way ANOVA (**b**) was used for statistical analyses

In a proof-of-concept approach, we next tested a library of 189 small compounds known to modulate apoptosis, survival, and proliferation in our CI-huVEC co-culture assay. After 6 days, the relative proportions of knockout alleles were quantified by amplicon deep sequencing (Fig. 6a). As expected, the knockout allele frequency in DMSO-treated co-cultures increased to 33.5%. Two compounds, isoalantolactone and NSC59984, suppressed the abnormal proliferation of mutant ECs by more than 80% (adjusted *p* value < 0.01; Fig. 6b). Knockout allele frequencies were 7.7% and 10.2%, respectively. NSC59984 had minimal effects on EC morphology and moderately reduced the viability of *CCM3*^+*/*+^ and *CCM3*^*−/−*^ CI-huVECs (Fig. 6c, d). Isoalantolactone induced an abnormal endothelial morphology and significant cell death. In contrast, GSK-872, which is a potent inhibitor of RIP kinase 3 (RIPK3), even enhanced mutant EC expansion (65.1%, *p*_adj_ < 0.0001; Fig. 6b, d). Interestingly, RIPK3 is a critical player in necroptosis, a regulated form of necrosis that allows programmed cell death even under apoptosis-deficient conditions [29, 30].

### p21- and ERK2-independent inhibition of abnormal mutant cell proliferation by NSC59984

**Fig. 7:**
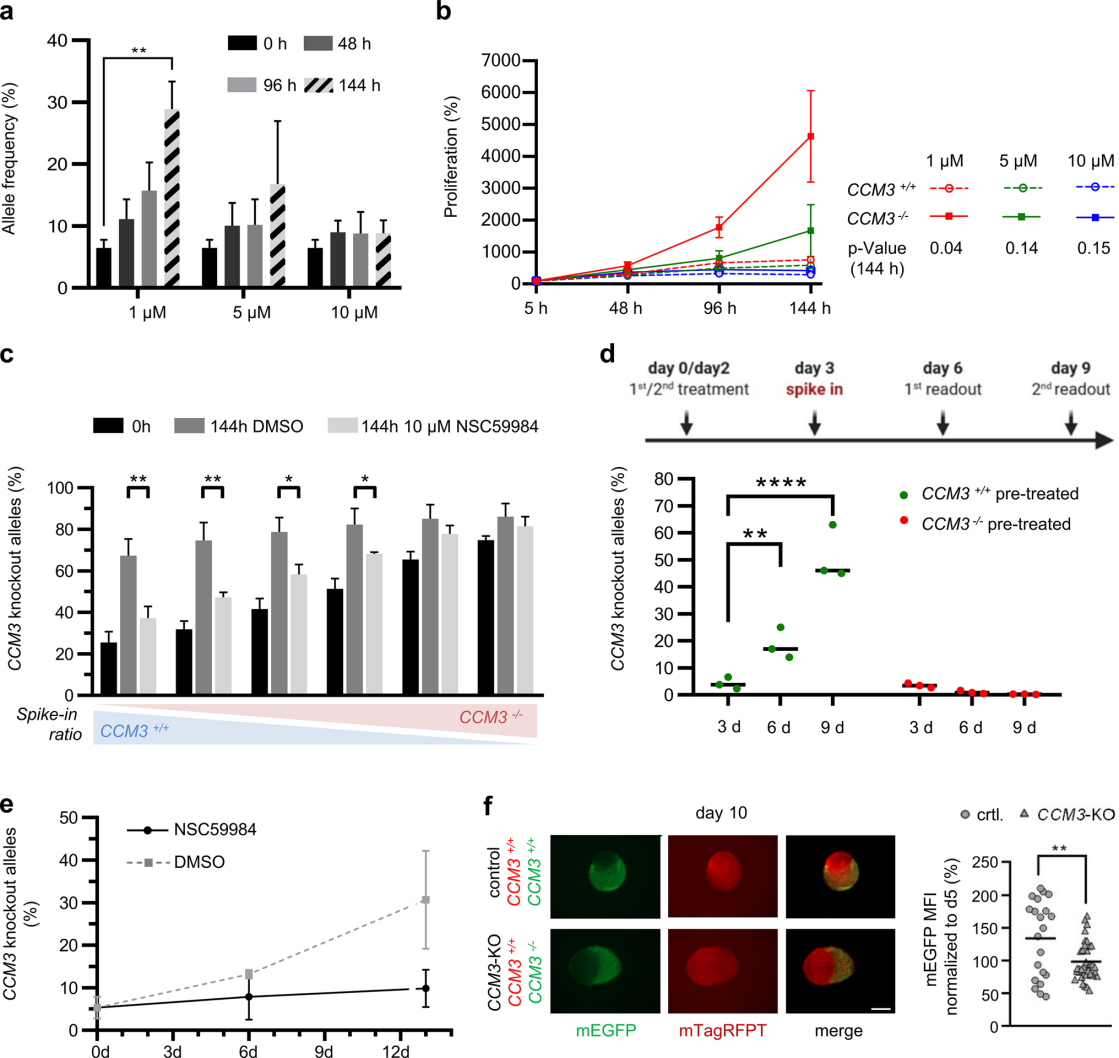
NSC59984 has cytostatic effects on *CCM3*^*−/−*^ ECs. **a**, **b** NSC59984 blocked the abnormal proliferation of *CCM3*^*−/−*^ CI-huVECs in co-culture in a dose-dependent manner and predominantly inhibited the proliferation of mutant ECs (*n* = 3 per group). **c** NSC59984 (10 µM) reduced the abnormal proliferation of mutant ECs even at high knockout:wild-type ratios [*n* = 3 per group; mutant allele frequency after seeding (0 h) from left to right: 25.5%, 31.9%, 41.6%, 51.4%, 65.4% and 74.8%]. **d** *CCM3*^*−/−*^ CI-huVECs were treated with NSC59984 prior to co-culture with untreated *CCM3*^+*/*+^ CI-huVECs (= *CCM3*^*−/−*^ pre-treated; red). As control, untreated *CCM3*^*−/−*^ CI-huVECs were co-cultured with *CCM3*^+*/*+^ CI-huVECs that had been pre-treated with NSC59984 (= *CCM3*^+*/*+^ pre-treated; green). Pre-treatment of *CCM3*^*−/−*^ CI-huVECs blocked their abnormal proliferation (red). In contrast, pre-treatment of *CCM3*^+*/*+^ led to an extremely strong proliferation of mutant ECs (*n* = 3 per group). **e** Endothelial mutant:wild-type co-cultures were treated with NSC59984 on day 0 and day 3, released from drug treatment on day 6, and cultured for another 7 days. NSC59984 retained its effect after treatment release. DMSO treated cultures served as control (*n* = 3 per group). **f** NSC59984 inhibited mutant EC proliferation in vascular organoids. *CCM3*^−/−^ AICS-0036 (mEGFP) and *CCM3*^+/+^ AICS-0054 (mTagRFPT) hiPSC-derived vascular networks were fused and differentiated to vascular organoids under NSC59984 treatment for 10 days (CCM3-KO). Images were acquired on days 5 and 10. Fusions of *CCM3*^+/+^ AICS-0036 and *CCM3*^+/+^ AICS-0054 hiPSC-derived vascular networks served as controls (scale: 500 µm). Statistical analysis was performed for 36 KO/WT fusion organoids differentiated in three independent biological replicates. 21 WT/WT fusion organoids were used as controls. Shown is the mean mEGFP fluorescence intensity on day 10 normalized to day 5. Data are presented as mean and SD (**a**, **b**, **c**, **e**) or as mean and single data points (**d**, **f**). Multiple comparison test (Fisher’s LSD; a), two-way ANOVA with multiple comparison test (**b**), multiple t-tests (**c**), two-way ANOVA with Dunnett’s multiple comparison test (**d**), and Student’s two-tailed *t* test (**f**) were used for statistical analyses: *P < 0.05, **P < 0.01, ****P < 0.001

**Fig. 8:**
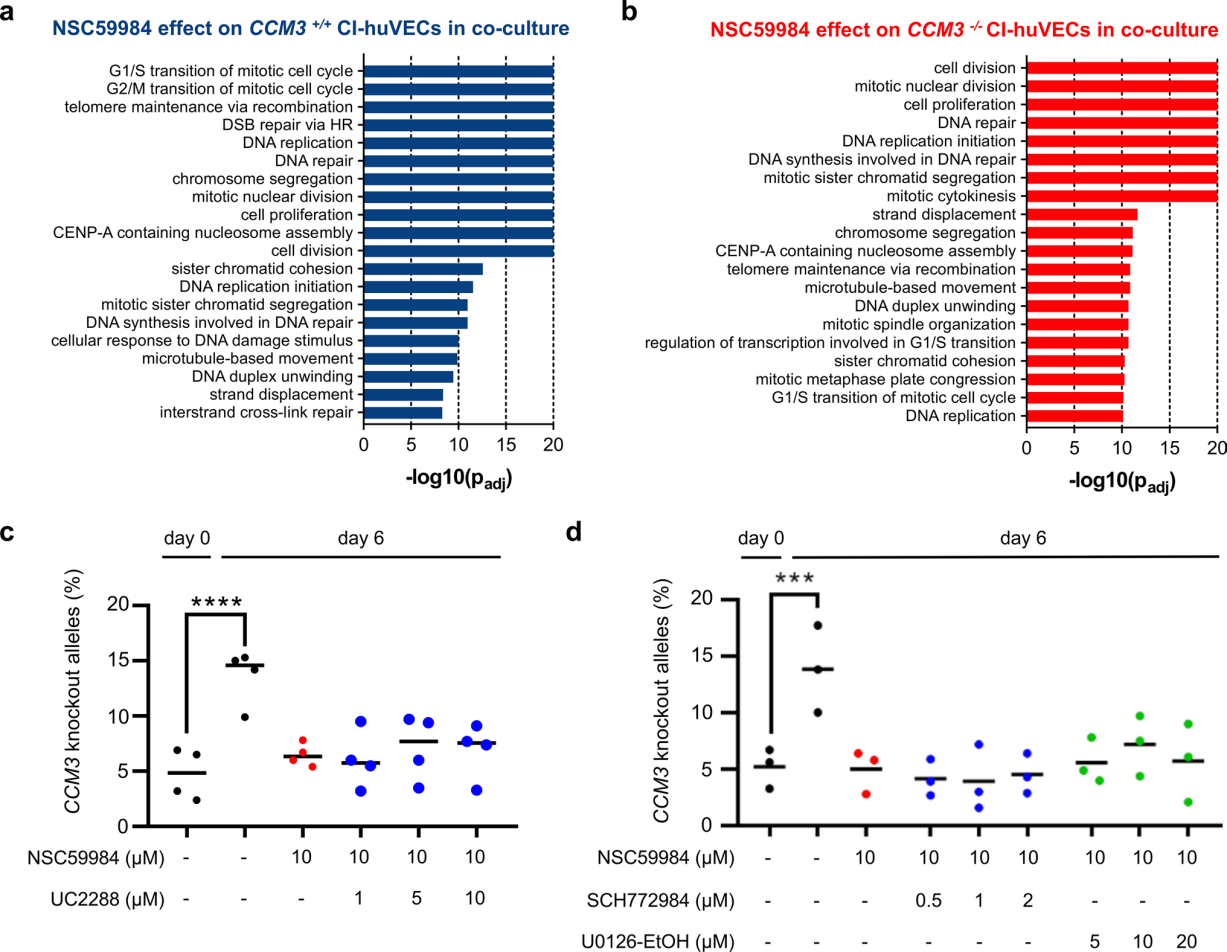
p21 and ERK-independent effect of NSC59984. **a**, **b** *CCM3*^*−/−*^ and *CCM3*^+*/*+^ CI-huVECs were co-cultured, treated with NSC59984 (10 µM), sorted and subjected to RNA sequencing. DMSO-treated co-cultures served as controls. Shown are the top 20 of significantly enriched biological process GO terms (*n* = 3 per group). **c**, **d** Co-cultures of *CCM3*^*−/−*^ and *CCM3*^+*/*+^ CI-huVECs were treated with NSC59984 (10 µM) and the p21 inhibitor UC2288 (*n* = 4 per group; **c**), the ERK1/2 inhibitor SCH772984 or the MEK1/2 inhibitor U0126-EtOH (*n* = 3 per group; **d**). Mutant allele frequencies were determined after 6 days. DMSO-treated co-cultures served as controls. Fisher exact test (**a**, **b**) and one-way ANOVA with multiple comparison test (**c**, **d**) were used for statistical analyses: ***P < 0.001; ****P < 0.0001. *p*_*adj*_ = adjusted *p* value

We decided to focus on the candidate NSC59984 in our further experiments and were able to demonstrate that it blocked the cancer-like proliferation of *CCM3*^*−/−*^ CI-huVECs in a concentration-dependent manner (Fig. 7a, b). NSC59984 inhibited mutant EC expansion even at high knockout:wild-type ratios (Fig. 7c). Pre-treatment of *CCM3*^*−/−*^ CI-huVECs with NSC59984 before co-culture completely abolished mutant EC expansion, and knockout alleles were hardly detectable in co-cultures after six days (Fig. 7d). Even after release from NSC59984 treatment, abnormal proliferation of *CCM3*^*−/−*^ CI-huVECs was blocked for at least another 7 days (Fig. 7e). An inhibitory effect on mutant cell proliferation was also observed when we fused hiPSC-derived *CCM3*^+*/*+^ and *CCM3*^*−/−*^ vascular networks and treated them with NSC59984 (Fig. 7f). This is consistent with our RNA sequencing data demonstrating a cytostatic effect of NSC59984 on mutant ECs. However, there were also side-effects. For instance, we found significant deregulation of proliferation-associated pathways in wild-type ECs (Fig. 8 a, b, Online Resources 9 and 10). NSC59984 has previously been shown to reactivate p53 signalling via p73 activation and ROS-ERK2-MDM2-dependent degradation of mutant p53 in cancer cells [19, 20]. In *CCM3*^*−/−*^ CI-huVECs we could neither detect a pathogenic *TP53* mutation nor significantly different p53 protein levels. Interestingly, we even found a slightly increased p53 transcription factor DNA binding activity in nuclear extracts of *CCM3*^−/−^ CI-huVECs which indicates that there might be a downstream block of p53 signaling (Online Resource 10). NSC59984 treatment of mutant ECs induced moderate upregulation of *CDKN1A* (p21), which is a target gene of p53, on mRNA level (Online Resource 10). However, p21 protein levels were unchanged and blocking p21 with UC2288 did also not revert the inhibitory effect of NSC59984 on mutant EC expansion in co-culture (Fig. 8c, Online Resource 10). We also addressed MAPK/ERK signaling in mutant ECs with protein array analyses and specific inhibitors but did not detect any significant deregulation either (Fig. 8d, Online Resource 10). Taken together, these results show that pharmacological inhibition of the abnormal proliferation of mutant ECs is possible. Nevertheless, side effects need to be addressed, and it remains to be clarified whether agents such as NSC59984 act through specific pathways in mutant ECs or have a general cytostatic effect on multiple signaling cascades.

## Discussion

CCM is not a genuine malignant disease. The lesions do not metastasize or grow invasively. However, there is growing evidence that cancer-like mechanisms play an important role in CCM pathogenesis [17, 18, 31]. In line with this concept, we here demonstrate that the presence of wild-type cells upregulates mutant EC proliferation and that this tumor-like behaviour can be blocked by treatment with the anti-cancer drug NSC59984. In addition, we show that gene expression in wild-type and mutant ECs differs in mono- and co-culture.

Previously, we have shown that *CCM3*^*−/−*^ ECs exhibit a reduced sensitivity to apoptotic signals in mono-culture [22]. Bringing together in vitro angiogenesis, endothelial co-culture and cell-based assays from cancer research, we now demonstrate that mutant ECs have an abnormally high clonogenic capacity but develop tumor-like growth characteristics only when co-cultured with wild-type ECs. This observation is highly relevant because mutant ECs (CCM^neg^) coexist in a mosaic state with heterozygous or wild-type ECs (CCM^pos^) in CCMs [15, 16]. Indeed, the number of CCM^neg^ cells within CCMs is relatively low. Despite some high-frequency mutations, somatic *CCM* variants are usually found with alternate allele frequencies of less than 15% in human CCM lesions [11–14, 16, 31]. Given that (1) only one somatic mutation has ever been identified in CCM tissues in addition to the preexisting germline variant, and (2) multiple somatic mutation events all leading to the same variant in different ECs are highly unlikely, solely expansion of the first CCM^neg^ EC can explain the endothelial mosaicism found in CCMs [16–18]. Furthermore, our finding that biallelic inactivation of *CCM3* did not induce abnormal proliferation of undifferentiated hiPSCs in co-culture with wild-type cells but specifically disturbed the balance between proliferation and cell death in vascular ECs supports the intriguing concept that *CCM* genes are not *bona fide* tumor suppressor genes but act as “*vascular suppressor genes*” [31]. This tumor-like vascular transformation seems to occur only under specific conditions and may be triggered in vivo by additional somatic gain-of-function mutations in *PIK3CA* which can act as a “*vascular oncogene*” [31].

The endothelial cell mosaicism observed in CCM tissues could also be reproduced in CRISPR’ed hiPSC-derived 3D blood vessel organoid cultures. CCM3^neg^ cells were not located in clusters but found in a mosaic pattern with CCM3^pos^ cells in CCM3^pos^/CCM3^neg^ vascular organoids. Thus, CCM3^neg^ ECs apparently need contact with CCM3^pos^ ECs and a supportive microenvironment. Interestingly, sophisticated in vivo studies in confetti reporter mice have also demonstrated that abnormal expansion of CCM3^neg^ ECs and incorporation of CCM^pos^ ECs are essential steps in CCM genesis [17, 18]. Targeted inhibition of CCM progenitor ECs, blocking the clonal expansion of mutant ECs and inhibiting the recruitment of neighboring CCM^pos^ ECs into a growing CCM lesion have, therefore, been proposed as therapeutic approaches [32].

Our data allow an exciting insight into the poorly understood interaction of CCM3^pos^ and CCM3^neg^ ECs. As a marker for CCM3^neg^ ECs, we used high expression levels of integrin *ß*4, which is known to form a heterodimer with integrin *α*6 and is primarily expressed on epithelial cells, endothelial cells, and Schwann cells [33]. Upregulation of *ITGB4* gene expression has previously been observed in human *CCM3*^*−/−*^ CI-huVECs ([34]; GEO data set: GSE137425), mouse *Ccm1*^*ECKO*^ brain microvascular endothelial cells ([35]; GSE85657), and cardiac tissue of *ccm2*^*m201*^ mutant zebrafish embryos ([36]; GSE64753). Although its functional significance in CCM disease remains to be elucidated, it is noteworthy that ITGB4 expression is increased on various cancer cells and is thought to be involved in the regulation of cancer stem cells [37–39]. RNA-Seq analysis of FACS-sorted CCM3^pos^ ITGB4^low^ and CCM3^neg^ ITGB4^high^ ECs demonstrated that chemokine-mediated signaling pathways are induced by direct co-culture. In particular, *CCL2*, *CX3CL1*, and *CCL7* were upregulated more than fourfold in CCM3^pos^ ECs. These genes encode for chemokines (CCL2/MCP-1; CX3CL1/Fractalkine; CCL7/ MCP-3) that modulate the immune response but have also frequently been found to be enriched in the tumor microenvironment [40–42]. Notably, it has been demonstrated that they can orchestrate the cross-talk between cancer and stromal cells, stimulate angiogenesis, and trigger proliferation [41, 43, 44]. Additionally, the CXC chemokine genes *CXCL2*, *CXCL3*, and *CXCL8* were upregulated more than twofold. CXCL2*,* CXCL3, and CXCL8/IL-8 belong to the group of CXC chemokines with a Glu-Leu-Arg sequence motif (ELR^+^ CXC chemokines) which are not only potent neutrophil chemoattractants but have also been reported to trigger angiogenesis and EC chemotaxis [45–47]. The results of our in vitro model are also consistent with recently published analyses in *Ccm3*^*iECKO*^ mice. Using an acute, “fast progression” and a chronic, “slow progression” model of CCM, the authors demonstrated that the expression of *Ccl2*, *Cxcl2*, *Cx3cl1* and other immune-related genes was significantly upregulated in brain microvascular ECs of *Ccm3*^*iECKO*^ mice [48]. In general, the proportion of cells with a *Ccm3* deletion is relatively low (< 15%) in the chronic model [48]. In the acute model, it is much higher (> 80%) but cells with *Ccm3* wild-type alleles are still present [17]. Our data now suggest that not only the mutant ECs themselves but also the wild-type ECs in contact with the mutant ECs upregulate immune-associated genes.

Taken together, these results strengthen the intriguing hypothesis that CCM3^pos^ ECs contribute to the formation of a supportive niche for CCM3^neg^ ECs and that the cancer-like nature of CCMs warrants testing of anti-cancer therapies. Two facts substantiate this concept: (1) NSC59984, a drug with anti-tumor activity [19], has a significant anti-proliferative effect on mutant ECs; and (2) ponatinib, a kinase inhibitor that is used to treat chronic myeloid leukemia and Ph^+^ acute lymphoblastic leukemia, has been reported to stop CCM formation and lesion growth in *Ccm1*^*iECKO*^ mice [49]. However, NSC59984 also induces growth arrest in wild-type ECs. Similarly, the kinase inhibitor ponatinib has a broad spectrum of side effects that would likely limit its therapeutic use in CCM, e.g., thrombocytopenia, abdominal pain, anemia, and even cardiac arrhythmias [50]. Thus, the identification of new drug candidates is required. Because of their side effects, cytotoxic agents might not be a real option for CCM patients who can have severe neurological complications but are also often asymptomatic. Modulating the microenvironment of CCMs instead would be an exciting approach. Several chemokine receptor inhibitors are currently under investigation in the context of solid tumors [51]. Our next-generation sequencing-based read-out system allows tracking mutant cell expansion in co-culture on a genetic level and will facilitate the testing of thousands of potential drug candidates. Such high-throughput screens may bring drug discovery in CCM research onto the next level.

In summary, there is increasing evidence that cancer-like mechanisms promote early CCM formation and lesion growth and that these mechanisms need to be addressed with new therapeutic approaches. In this context, it is essential to focus not only on CCM^neg^ but also on CCM^pos^ cells and to clarify their role in CCM pathogenesis.

### Supplementary Information

Below is the link to the electronic supplementary material.

Supplementary file1 (PDF 1759 KB)

Supplementary file2 (XLSX 18 KB)

Supplementary file3 (XLSX 764 KB)

## Data Availability

All relevant data are included in the published article or its supplementary files.

## Abbreviations

ADS: Amplicon deep sequencing
CCM: Cerebral cavernous malformations
CCM^pos^: Heterozygous or wild-type ECs
CCM^neg^: Mutant ECs
CI-huVECs: Constitutive immortalized human umbilical vein endothelial cells
EC: Endothelial cells
ECGM: Endothelial cell growth medium
FCS: Fetal calf serum
hBMEC-like cells: Human brain microvascular endothelial-like cells
hESFM: Human endothelial serum-free medium
hiPSC: Human induced pluripotent stem cell
ITGB4: Integrin β4
PFA: Paraformaldehyde
RNP: Ribonucleoprotein
SD: Standard deviation

## References

[1] Batra S, Lin D, Recinos PF, Zhang J, Rigamonti D (2009). Cavernous malformations: natural history, diagnosis and treatment. Nat Rev Neurol.

[2] Akers A, Al-Shahi Salman R, Awad AI, Dahlem K, Flemming K, Hart B, Kim H, Jusue-Torres I, Kondziolka D, Lee C, Morrison L, Rigamonti D, Rebeiz T, Tournier-Lasserve E, Waggoner D, Whitehead K (2017). Synopsis of guidelines for the clinical management of cerebral cavernous malformations: consensus recommendations based on systematic literature review by the angioma alliance scientific advisory board clinical experts panel. Neurosurgery.

[3] Spiegler S, Rath M, Paperlein C, Felbor U (2018). Cerebral cavernous malformations: an update on prevalence, molecular genetic analyses, and genetic counselling. Mol Syndromol.

[4] Shenkar R, Shi C, Rebeiz T, Stockton RA, McDonald DA, Mikati AG, Zhang L, Austin C, Akers AL, Gallione CJ, Rorrer A, Gunel M, Min W, De Souza JM, Lee C, Marchuk DA, Awad IA (2015). Exceptional aggressiveness of cerebral cavernous malformation disease associated with *PDCD10* mutations. Genet Med.

[5] Spiegler S, Najm J, Liu J, Gkalympoudis S, Schröder W, Borck G, Brockmann K, Elbracht M, Fauth C, Ferbert A, Freudenberg L, Grasshoff U, Hellenbroich Y, Henn W, Hoffjan S, Huning I, Korenke GC, Kroisel PM, Kunstmann E, Mair M, Munk-Schulenburg S, Nikoubashman O, Pauli S, Rudnik-Schoneborn S, Sudholt I, Sure U, Tinschert S, Wiednig M, Zoll B, Ginsberg MH, Felbor U (2014). High mutation detection rates in cerebral cavernous malformation upon stringent inclusion criteria: one-third of probands are minors. Mol Genet Genomic Med.

[6] He Y, Zhang H, Yu L, Gunel M, Boggon TJ, Chen H, Min W (2010). Stabilization of VEGFR2 signaling by cerebral cavernous malformation 3 is critical for vascular development. Sci Signal.

[7] Zeineddine HA, Girard R, Saadat L, Shen L, Lightle R, Moore T, Cao Y, Hobson N, Shenkar R, Avner K, Chaudager K, Koskimäki J, Polster SP, Fam MD, Shi C, Lopez-Ramirez MA, Tang AT, Gallione C, Kahn ML, Ginsberg M, Marchuk DA, Awad IA (2019). Phenotypic characterization of murine models of cerebral cavernous malformations. Lab Invest.

[8] Louvi A, Chen L, Two AM, Zhang H, Min W, Gunel M (2011). Loss of *cerebral cavernous malformation 3* (*Ccm3*) in neuroglia leads to CCM and vascular pathology. Proc Natl Acad Sci U S A.

[9] Wang K, Zhang H, He Y, Jiang Q, Tanaka Y, Park IH, Pober JS, Min W, Zhou HJ (2020). Mural cell-specific deletion of cerebral cavernous malformation 3 in the brain induces cerebral cavernous malformations. Arterioscler Thromb Vasc Biol.

[10] Lopez-Ramirez MA, Lai CC, Soliman SI, Hale P, Pham A, Estrada EJ, McCurdy S, Girard R, Verma R, Moore T, Lightle R, Hobson N, Shenkar R, Poulsen O, Haddad GG, Daneman R, Gongol B, Sun H, Lagarrigue F, Awad IA, Ginsberg MH (2021). Astrocytes propel neurovascular dysfunction during cerebral cavernous malformation lesion formation. J Clin Invest.

[11] Gault J, Shenkar R, Recksiek P, Awad IA (2005). Biallelic somatic and germ line *CCM1* truncating mutations in a cerebral cavernous malformation lesion. Stroke.

[12] Gault J, Awad IA, Recksiek P, Shenkar R, Breeze R, Handler M, Kleinschmidt-DeMasters BK (2009). Cerebral cavernous malformations: somatic mutations in vascular endothelial cells. Neurosurgery.

[13] Akers AL, Johnson E, Steinberg GK, Zabramski JM, Marchuk DA (2009). Biallelic somatic and germline mutations in cerebral cavernous malformations (CCMs): evidence for a two-hit mechanism of CCM pathogenesis. Hum Mol Genet.

[14] McDonald DA, Shi C, Shenkar R, Gallione CJ, Akers AL, Li S, De Castro N, Berg MJ, Corcoran DL, Awad IA, Marchuk DA (2014). Lesions from patients with sporadic cerebral cavernous malformations harbor somatic mutations in the CCM genes: evidence for a common biochemical pathway for CCM pathogenesis. Hum Mol Genet.

[15] Pagenstecher A, Stahl S, Sure U, Felbor U (2009). A two-hit mechanism causes cerebral cavernous malformations: complete inactivation of CCM1, CCM2 or CCM3 in affected endothelial cells. Hum Mol Genet.

[16] Rath M, Pagenstecher A, Hoischen A, Felbor U (2020). Postzygotic mosaicism in cerebral cavernous malformation. J Med Genet.

[17] Malinverno M, Maderna C, Abu Taha A, Corada M, Orsenigo F, Valentino M, Pisati F, Fusco C, Graziano P, Giannotta M, Yu QC, Zeng YA, Lampugnani MG, Magnusson PU, Dejana E (2019). Endothelial cell clonal expansion in the development of cerebral cavernous malformations. Nat Commun.

[18] Detter MR, Snellings DA, Marchuk DA (2018). Cerebral cavernous malformations develop through clonal expansion of mutant endothelial cells. Circ Res.

[19] Zhang S, Zhou L, Hong B, van den Heuvel AP, Prabhu VV, Warfel NA, Kline CL, Dicker DT, Kopelovich L, El-Deiry WS (2015). Small-molecule NSC59984 restores p53 pathway signaling and antitumor effects against colorectal cancer via p73 activation and degradation of mutant p53. Cancer Res.

[20] Zhang S, Zhou L, El-Deiry WS (2022). Small-molecule NSC59984 induces mutant p53 degradation through a ROS-ERK2-MDM2 Axis in cancer cells. Mol Cancer Res.

[21] Lipps C, Klein F, Wahlicht T, Seiffert V, Butueva M, Zauers J, Truschel T, Luckner M, Köster M, MacLeod R, Pezoldt J, Hühn J, Yuan Q, Müller PP, Kempf H, Zweigerdt R, Dittrich-Breiholz O, Pufe T, Beckmann R, Drescher W, Riancho J, Sanudo C, Korff T, Opalka B, Rebmann V, Göthert JR, Alves PM, Ott M, Schucht R, Hauser H, Wirth D, May T (2018). Expansion of functional personalized cells with specific transgene combinations. Nat Commun.

[22] Schwefel K, Spiegler S, Ameling S, Much CD, Pilz RA, Otto O, Völker U, Felbor U, Rath M (2019). Biallelic *CCM3* mutations cause a clonogenic survival advantage and endothelial cell stiffening. J Cell Mol Med.

[23] Schwefel K, Spiegler S, Much CD, Felbor U, Rath M (2020). CRISPR/Cas9-mediated generation of human endothelial cell knockout models of CCM disease. Methods Mol Biol.

[24] Neal EH, Marinelli NA, Shi Y, McClatchey PM, Balotin KM, Gullett DR, Hagerla KA, Bowman AB, Ess KC, Wikswo JP, Lippmann ES (2019). A simplified, fully defined differentiation scheme for producing blood-brain barrier endothelial cells from human iPSCs. Stem Cell Reports.

[25] Wimmer RA, Leopoldi A, Aichinger M, Kerjaschki D, Penninger JM (2019). Generation of blood vessel organoids from human pluripotent stem cells. Nat Protoc.

[26] Franken NA, Rodermond HM, Stap J, Haveman J, van Bree C (2006). Clonogenic assay of cells in vitro. Nat Protoc.

[27] Nowak-Sliwinska P, Alitalo K, Allen E, Anisimov A, Aplin AC, Auerbach R, Augustin HG, Bates DO, van Beijnum JR, Bender RHF, Bergers G, Bikfalvi A, Bischoff J, Böck BC, Brooks PC, Bussolino F, Cakir B, Carmeliet P, Castranova D, Cimpean AM, Cleaver O, Coukos G, Davis GE, De Palma M, Dimberg A, Dings RPM, Djonov V, Dudley AC, Dufton NP, Fendt SM, Ferrara N, Fruttiger M, Fukumura D, Ghesquiere B, Gong Y, Griffin RJ, Harris AL, Hughes CCW, Hultgren NW, Iruela-Arispe ML, Irving M, Jain RK, Kalluri R, Kalucka J, Kerbel RS, Kitajewski J, Klaassen I, Kleinmann HK, Koolwijk P, Kuczynski E, Kwak BR, Marien K, Melero-Martin JM, Munn LL, Nicosia RF, Noel A, Nurro J, Olsson AK, Petrova TV, Pietras K, Pili R, Pollard JW, Post MJ, Quax PHA, Rabinovich GA, Raica M, Randi AM, Ribatti D, Ruegg C, Schlingemann RO, Schulte-Merker S, Smith LEH, Song JW, Stacker SA, Stalin J, Stratman AN, Van de Velde M, van Hinsbergh VWM, Vermeulen PB, Waltenberger J, Weinstein BM, Xin H, Yetkin-Arik B, Yla-Herttuala S, Yoder MC, Griffioen AW (2018). Consensus guidelines for the use and interpretation of angiogenesis assays. Angiogenesis.

[28] Wimmer RA, Leopoldi A, Aichinger M, Wick N, Hantusch B, Novatchkova M, Taubenschmid J, Hämmerle M, Esk C, Bagley JA, Lindenhofer D, Chen G, Boehm M, Agu CA, Yang F, Fu B, Zuber J, Knoblich JA, Kerjaschki D, Penninger JM (2019). Human blood vessel organoids as a model of diabetic vasculopathy. Nature.

[29] Shan B, Pan H, Najafov A, Yuan J (2018). Necroptosis in development and diseases. Genes Dev.

[30] Kim C, Pasparakis M (2014). RIP kinase 3 in necroptosis: does it take two or more to kill?. Cell Death Differ.

[31] Ren AA, Snellings DA, Su YS, Hong CC, Castro M, Tang AT, Detter MR, Hobson N, Girard R, Romanos S, Lightle R, Moore T, Shenkar R, Benavides C, Beaman MM, Müller-Fielitz H, Chen M, Mericko P, Yang J, Sung DC, Lawton MT, Ruppert JM, Schwaninger M, Körbelin J, Potente M, Awad IA, Marchuk DA, Kahn ML (2021). PIK3CA and CCM mutations fuel cavernomas through a cancer-like mechanism. Nature.

[32] Abdelilah-Seyfried S, Tournier-Lasserve E, Derry WB (2020). Blocking signalopathic events to treat cerebral cavernous malformations. Trends Mol Med.

[33] Dusek RL, Jones JCR, Green KJ, Lennarz WJ, Lane MD (2004). Desmosomes and Hemidesmosomes. Encyclopedia of Biological Chemistry.

[34] Schwefel K, Spiegler S, Kirchmaier BC, Dellweg PKE, Much CD, Pane-Farre J, Strom TM, Riedel K, Felbor U, Rath M (2020). Fibronectin rescues aberrant phenotype of endothelial cells lacking either CCM1, CCM2 or CCM3. FASEB J.

[35] Lopez-Ramirez MA, Fonseca G, Zeineddine HA, Girard R, Moore T, Pham A, Cao Y, Shenkar R, de Kreuk BJ, Lagarrigue F, Lawler J, Glass CK, Awad IA, Ginsberg MH (2017). Thrombospondin1 (TSP1) replacement prevents cerebral cavernous malformations. J Exp Med.

[36] Renz M, Otten C, Faurobert E, Rudolph F, Zhu Y, Boulday G, Duchene J, Mickoleit M, Dietrich AC, Ramspacher C, Steed E, Manet-Dupe S, Benz A, Hassel D, Vermot J, Huisken J, Tournier-Lasserve E, Felbor U, Sure U, Albiges-Rizo C, Abdelilah-Seyfried S (2015). Regulation of beta1 integrin-Klf2-mediated angiogenesis by CCM proteins. Dev Cell.

[37] Ruan S, Lin M, Zhu Y, Lum L, Thakur A, Jin R, Shao W, Zhang Y, Hu Y, Huang S, Hurt EM, Chang AE, Wicha MS, Li Q (2020). Integrin beta4-targeted cancer immunotherapies inhibit tumor growth and decrease metastasis. Cancer Res.

[38] Bierie B, Pierce SE, Kroeger C, Stover DG, Pattabiraman DR, Thiru P, Liu Donaher J, Reinhardt F, Chaffer CL, Keckesova Z, Weinberg RA (2017). Integrin-beta4 identifies cancer stem cell-enriched populations of partially mesenchymal carcinoma cells. Proc Natl Acad Sci U S A.

[39] Ma B, Zhang L, Zou Y, He R, Wu Q, Han C, Zhang B (2019). Reciprocal regulation of integrin beta4 and KLF4 promotes gliomagenesis through maintaining cancer stem cell traits. J Exp Clin Cancer Res.

[40] Lee YS, Cho YB (2020). CCL7 Signaling in the tumor microenvironment. Adv Exp Med Biol.

[41] Tsuyada A, Chow A, Wu J, Somlo G, Chu P, Loera S, Luu T, Li AX, Wu X, Ye W, Chen S, Zhou W, Yu Y, Wang YZ, Ren X, Li H, Scherle P, Kuroki Y, Wang SE (2012). CCL2 mediates cross-talk between cancer cells and stromal fibroblasts that regulates breast cancer stem cells. Cancer Res.

[42] Rivas-Fuentes S, Salgado-Aguayo A, Arratia-Quijada J, Gorocica-Rosete P (2021). Regulation and biological functions of the CX3CL1-CX3CR1 axis and its relevance in solid cancer: A mini-review. J Cancer.

[43] Rajaram M, Li J, Egeblad M, Powers RS (2013). System-wide analysis reveals a complex network of tumor-fibroblast interactions involved in tumorigenicity. PLoS Genet.

[44] Lee SJ, Namkoong S, Kim YM, Kim CK, Lee H, Ha KS, Chung HT, Kwon YG, Kim YM (2006). Fractalkine stimulates angiogenesis by activating the Raf-1/MEK/ERK- and PI3K/Akt/eNOS-dependent signal pathways. Am J Physiol Heart Circ Physiol.

[45] Addison CL, Daniel TO, Burdick MD, Liu H, Ehlert JE, Xue YY, Buechi L, Walz A, Richmond A, Strieter RM (2000). The CXC chemokine receptor 2, CXCR2, is the putative receptor for ELR+ CXC chemokine-induced angiogenic activity. J Immunol.

[46] Rollins BJ (1997). Chemokines. Blood.

[47] Strieter RM, Burdick MD, Gomperts BN, Belperio JA, Keane MP (2005). CXC chemokines in angiogenesis. Cytokine Growth Factor Rev.

[48] Yau ACY, Globisch MA, Onyeogaziri FC, Conze LL, Smith R, Jauhiainen S, Corada M, Orsenigo F, Huang H, Herre M, Olsson AK, Malinverno M, Sundell V, Rezai Jahromi B, Niemelä M, Laakso A, Garlanda C, Mantovani A, Lampugnani MG, Dejana E, Magnusson PU (2022). Inflammation and neutrophil extracellular traps in cerebral cavernous malformation. Cell Mol Life Sci.

[49] Choi JP, Wang R, Yang X, Wang X, Wang L, Ting KK, Foley M, Cogger V, Yang Z, Liu F, Han Z, Liu R, Baell J, Zheng X (2018). Ponatinib (AP24534) inhibits MEKK3-KLF signaling and prevents formation and progression of cerebral cavernous malformations. Sci Adv.

[50] Chan O, Talati C, Isenalumhe L, Shams S, Nodzon L, Fradley M, Sweet K, Pinilla-Ibarz J (2020). Side-effects profile and outcomes of ponatinib in the treatment of chronic myeloid leukemia. Blood Adv.

[51] Mollica Poeta V, Massara M, Capucetti A, Bonecchi R (2019). Chemokines and chemokine receptors: new targets for cancer immunotherapy. Front Immunol.

