# Supplementary material for: Contact-dependent signaling triggers tumor-like proliferation of CCM3 knockout endothelial cells in co-culture with wild-type cells
Source: Cell Mol Life Sci. 2022 Jun 4;79(6):340. doi: 10.1007/s00018-022-04355-6 (PMC9166869; doi:10.1007/s00018-022-04355-6)
Supplement: Supplementary file 1 — Supplementary file1 (PDF 1759 KB) [file 18_2022_4355_MOESM1_ESM.pdf]

ONLINE RESOURCES

Contact-dependent signaling triggers tumor-like proliferation of *CCM3* knockout endothelial cells in co-culture with wild-type cells

Authors:

Matthias Rath[1,§,*]

Konrad Schwefel[1;§]

Matteo Malinverno[2]

Dariush Skowronek[1]

Alexandra Leopoldi[3]

Robin A. Pilz[1]

Doreen Biedenweg[4]

Sander Bekeschus[5]

Josef M. Penninger[3,6]

Elisabetta Dejana[2,7]

Ute Felbor[1]

Affiliations:

[1] Department of Human Genetics, University Medicine Greifswald and Interfaculty Institute of Genetics and Functional Genomics, University of Greifswald, Greifswald, Germany.

[2] Vascular Biology Unit, FIRC Institute of Molecular Oncology Foundation (IFOM), Milan, Italy.

[3] Institute of Molecular Biotechnology of the Austrian Academy of Sciences, Vienna, Austria.

[4] Centre for Innovation Competence - Humoral Immune Reactions in Cardiovascular Diseases, University of Greifswald, Greifswald, Germany.

[5] ZIK plasmatis, Leibniz Institute for Plasma Science and Technology (INP), Greifswald, Germany.

[6] Department of Medical Genetics, Life Sciences Institute, University of British Columbia, Vancouver, Canada.

[7] Department of Immunology, Genetics and Pathology, Uppsala University, Uppsala, Sweden.

[§] Both authors contributed equally

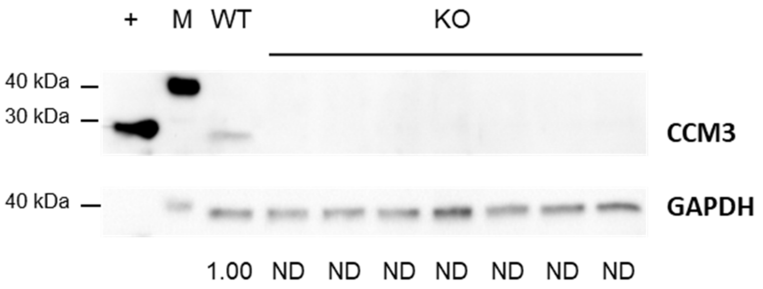

Online Resource 1 Verification of CCM3 knockout in CI-huVECs. Western Blot results are given for one wild-type CI-huVEC (WT) and seven *CCM3*$^{-/-}$ CI-huVEC cell lines (KO). No CCM3 protein was detected in the KO cell lines. Pure His-tagged CCM3 protein was used as a positive control (+). ND = not detected

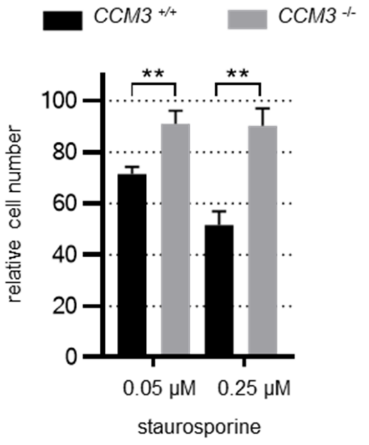

Online Resource 2 Increased cell survival rates of *CCM3*$^{-/-}$ CI-huVECs under staurosporine stress. *CCM3*$^{+/+}$ and *CCM3*$^{-/-}$ CI-huVECs were treated with either 0.05 µM or 0.25 µM staurosporine to induce apoptotic cell death. After 24 hours, the number of cells per mm^2 was determined and normalized to the initial cell count (n = 3 per genotype). Students two-tailed *t* tests were used for statistical analyses: **P<0.01

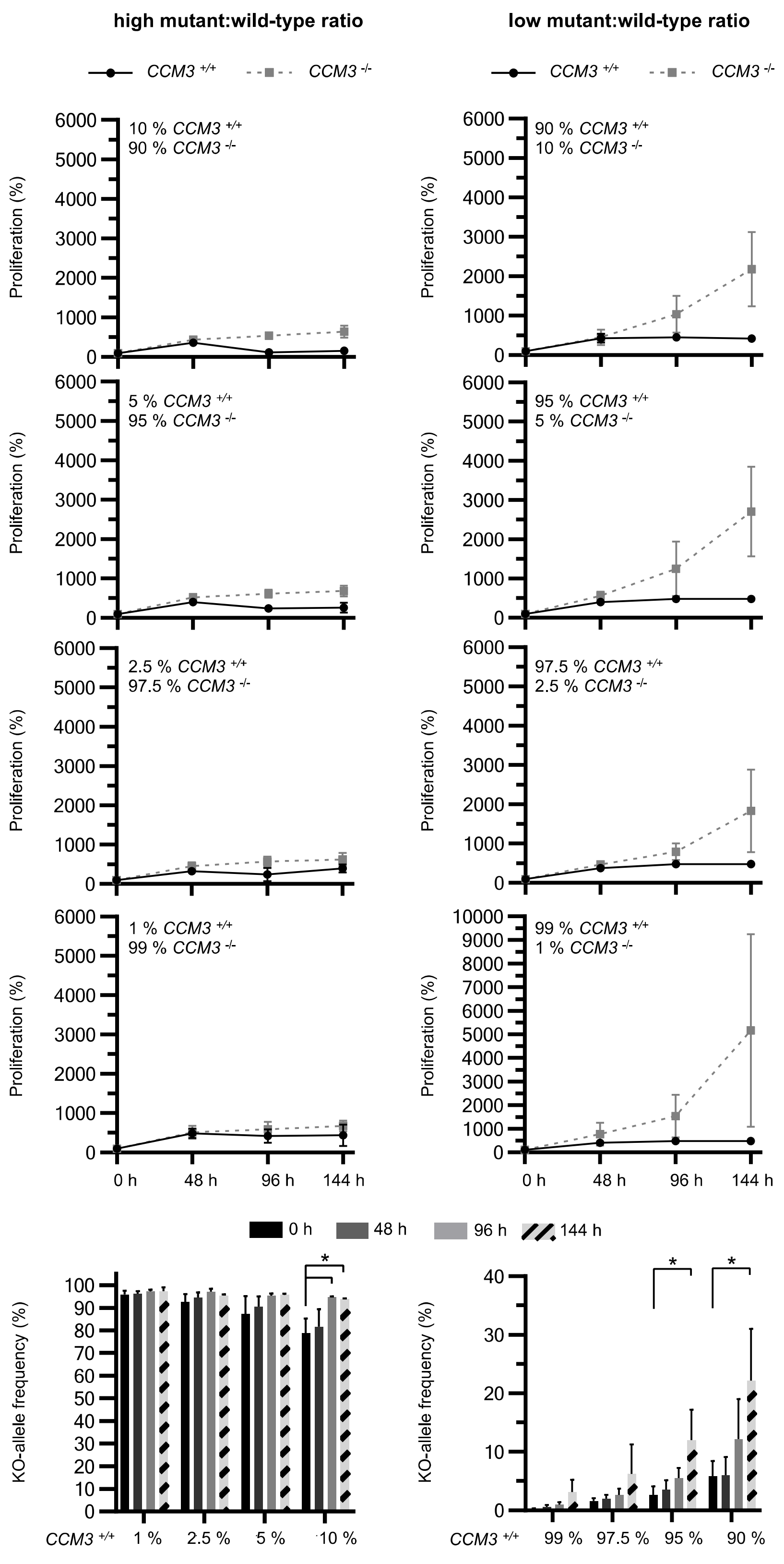
high mutant:wild-type ratio
low mutant:wild-type ratio
CCM3 +/+
CCM3 -/-
10 % CCM3 +/+
90 % CCM3 -/-
5 % CCM3 +/+
95 % CCM3 -/-
2.5 % CCM3 +/+
97.5 % CCM3 -/-
1 % CCM3 +/+
99 % CCM3 -/-
90 % CCM3 +/+
10 % CCM3 -/-
95 % CCM3 +/+
5 % CCM3 -/-
97.5 % CCM3 +/+
2.5 % CCM3 -/-
99 % CCM3 +/+
1 % CCM3 -/-
Proliferation (%)
0 h
48 h
96 h
144 h
KO-allele frequency (%)
CCM3 +/+
CCM3 -/-
1 %
2.5 %
5 %
10 %
99 %
97.5 %
95 %
90 %

Online Resource 4 *CCM3*$^{-/-}$ CI-huVECs demonstrate increased proliferative activity only in co-cultures with low mutant:wild-type ratios. *CCM3*$^{-/-}$ CI-huVECs were co-cultured with *CCM3*$^{+/+}$ CI-huVECs with high (left subpanels) or low (right subpanels) mutant:wild-type ratios, respectively (n = 3 per condition). The ratios are given in the upper left corner of each subpanel. Mutant allele frequencies were determined by amplicon deep sequencing. Cell counts were quantified with the CyQUANT cell proliferation assays. The proliferation rates of *CCM3*$^{-/-}$ and *CCM3*$^{+/+}$ CI-huVECs were calculated by combining the mutant allele frequencies and the cell numbers in co-culture at the indicated time points. Students two-tailed *t* tests were used for statistical analyses: *P<0.05

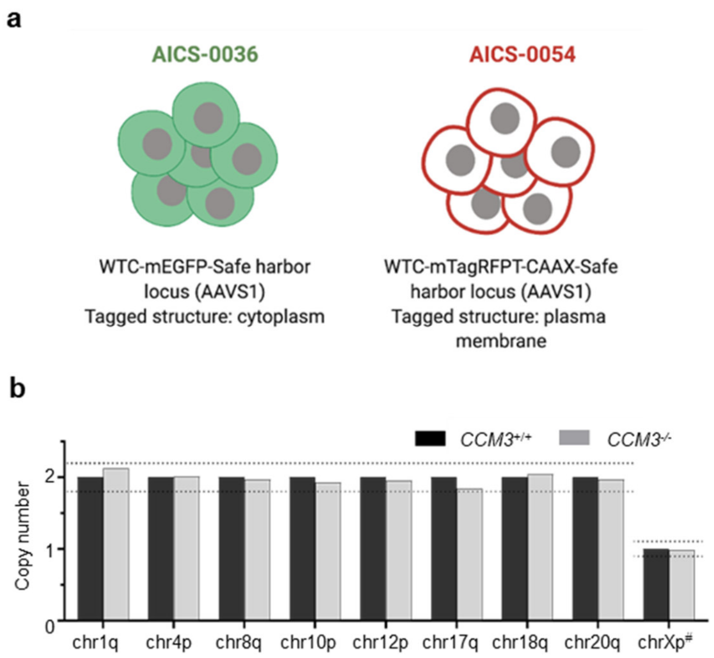

Online Resource 5 AICS-0036 and AICS-0054 hiPSCs. **a** AICS-0036 hiPSCs are characterized by cytoplasmic mEGFP expression. AICS-0054 hiPSCs with an mTagRFP-tag localized to the plasma membrane were used as controls. **b** The presence of the most common chromosomal abnormalities was excluded in *CCM3*$^{-/-}$ AICS-0036 hiPSC. Shown is a representative result for one *CCM3*$^{-/-}$ AICS-0036 hiPSC clone. * The parental hiPSC line (WTC/AICS-0) had been established from fibroblasts of a male donor. Therefore, the copy number for Xp is 1.

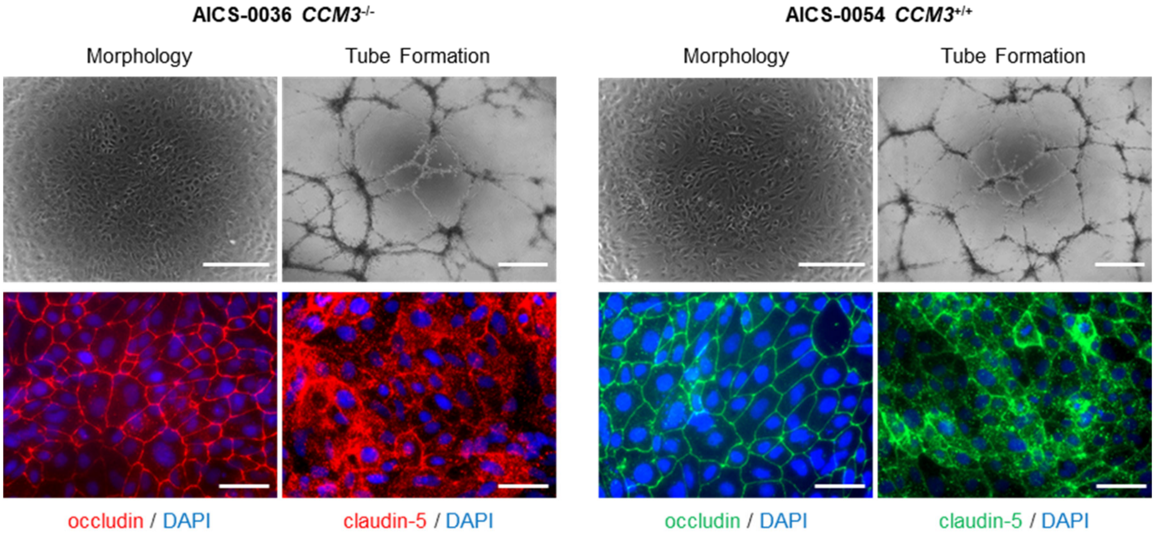

Online Resource 6 hiPSC-derived hBMEC-like cells express tight junction proteins and can form capillary-like structures. Representative images of the morphology (upper left panel, scale: 200 µm), tube formation assay (upper right panel, scale: 500 µm), and the immunofluorescence analyses of the tight junction proteins occludin and claudin-5 (lower panels, scale: 50 µm) for AICS-0036 *CCM3*$^{-/-}$ and AICS-0054 *CCM3*$^{+/+}$ derived hBMEC-like cells. Brightness was adjusted for occludin (AICS-0054 *CCM3*$^{+/+}$ derived hBMEC-like cells) for clarity.

Online Resource 7 Deregulated *ITGB4* gene expression in different CCM models. Listed p-values were adjusted for multiple testing (adj. p-value) by the authors. For Chernaya et. al 2018 differential gene expression was calculated using GFOLD (0.01), no p-values were included in the datasets.

Gen	Model	Strategy	Analysis	*ITGB4* log2FC	adj. p-value	Reference
CCM3	CI-huVEC	knockout	RNA-seq	4.28	2.57E-44	**Schwefel et al. 2020**
CCM1	BMEC	knockout	RNA-seq	2.50	6.07E-23	**Lopez-Ramirez et al. 2017**
CCM2	zebrafish	knockout	RNA-microarray	3.99	0.0104	**Renz et al. 2015**
CCM3	HCAEC	knockdown passage 7	RNA-microarray	1.96	0.0050	**Guerrero et al. 2015**
CCM3	HCAEC	knockdown passage 11		2.81	0.000014	
CCM1	HBMVEC	knockdown	RNA-seq	3.66	0.00005	**Abou-Fadel et al. 2019**
CCM2	HBMVEC	knockdown		0.09	0.00005	
CCM1	zebrafish	knockout		0.96	0.3872	
CCM2	zebrafish	knockout		-2.71	0.0031	
CCM1	HUVEC	knockdown	RNA-seq	3.46		**Chernaya et al. 2018**
CCM2	HUVEC			3.48		
CCM3	HUVEC			1.71		
CCM1	HUVEC	overexpression	RNA-microarray	-0.56	0.0039	**Wüstehube et al. 2010**

CI-huVEC = immortalized human umbilical vein endothelial cells
BMEC = human blood-brain barrier endothelial cells
HCAEC = human coronary artery endothelial cells
HBMVEC = human brain microvascular endothelial cells
HUVEC = Human umbilical vein endothelial cells

1. Schwefel K, Spiegler S, Kirchmaier BC, Dellweg PKE, Much CD, Pané-Farré J, et al. Fibronectin rescues aberrant phenotype of endothelial cells lacking either CCM1, CCM2 or CCM3. The FASEB Journal. 2020.
2. Lopez-Ramirez MA, Fonseca G, Zeineddine HA, Girard R, Moore T, Pham A, et al. Thrombospondin1 (TSP1) replacement prevents cerebral cavernous malformations. J Exp Med. 2017;214(11):3331-46.
3. Renz M, Otten C, Faurobert E, Rudolph F, Zhu Y, Boulday G, et al. Regulation of beta1 integrin-Klf2-mediated angiogenesis by CCM proteins. Dev Cell. 2015;32(2):181-90.
4. Guerrero A, Iglesias C, Raguz S, Floridia E, Gil J, Pombo CM, et al. The cerebral cavernous malformation 3 gene is necessary for senescence induction. Aging Cell. 2015;14(2):274-83.
5. Abou-Fadel J, Vasquez M, Grajeda B, Ellis C, Zhang J. Systems-wide analysis unravels the new roles of CCM signal complex (CSC). Heliyon. 2019;5(12):e02899.
6. Chernaya O, Zhurikhina A, Hladyshau S, Pilcher W, Young KM, Ortner J, et al. Biomechanics of Endothelial Tubule Formation Differentially Modulated by Cerebral Cavernous Malformation Proteins. iScience. 2018;9:347-58.
7. Wüstehube J, Bartol A, Liebler SS, Brutsch R, Zhu Y, Felbor U, et al. Cerebral cavernous malformation protein CCM1 inhibits sprouting angiogenesis by activating DELTA-NOTCH signaling. Proc Natl Acad Sci U S A. 2010;107(28):12640-5.

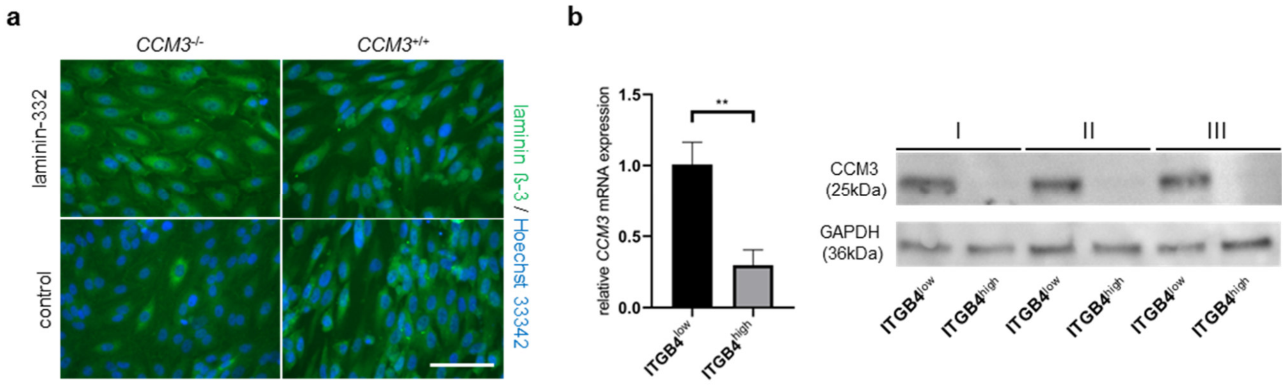

Online Resource 8 a *CCM3*$^{-/-}$ CI-huVECs show increased membrane-bound laminin on laminin-332-coated plates. Representative images of *CCM3*$^{-/-}$ and *CCM3*$^{+/+}$ CI-huVECs cultured on laminin-332-coated and non-coated plates (control) for 6 days (scale: 50 µm). **b** High purity of sorted ITGB4^{high} CCM3^{neg} and ITGB4^{low} CCM3^{pos} CI-huVEC populations. Western blot analysis verified complete CCM3 knockout in sorted ITGB4^{high} cells (n = 3 per condition). *CCM3* transcript levels were also significantly reduced in ITGB4^{high} CCM3^{neg} cells (n = 3 per condition). Students two-tailed *t* tests (b) were used for statistical analyses: **P<0.01

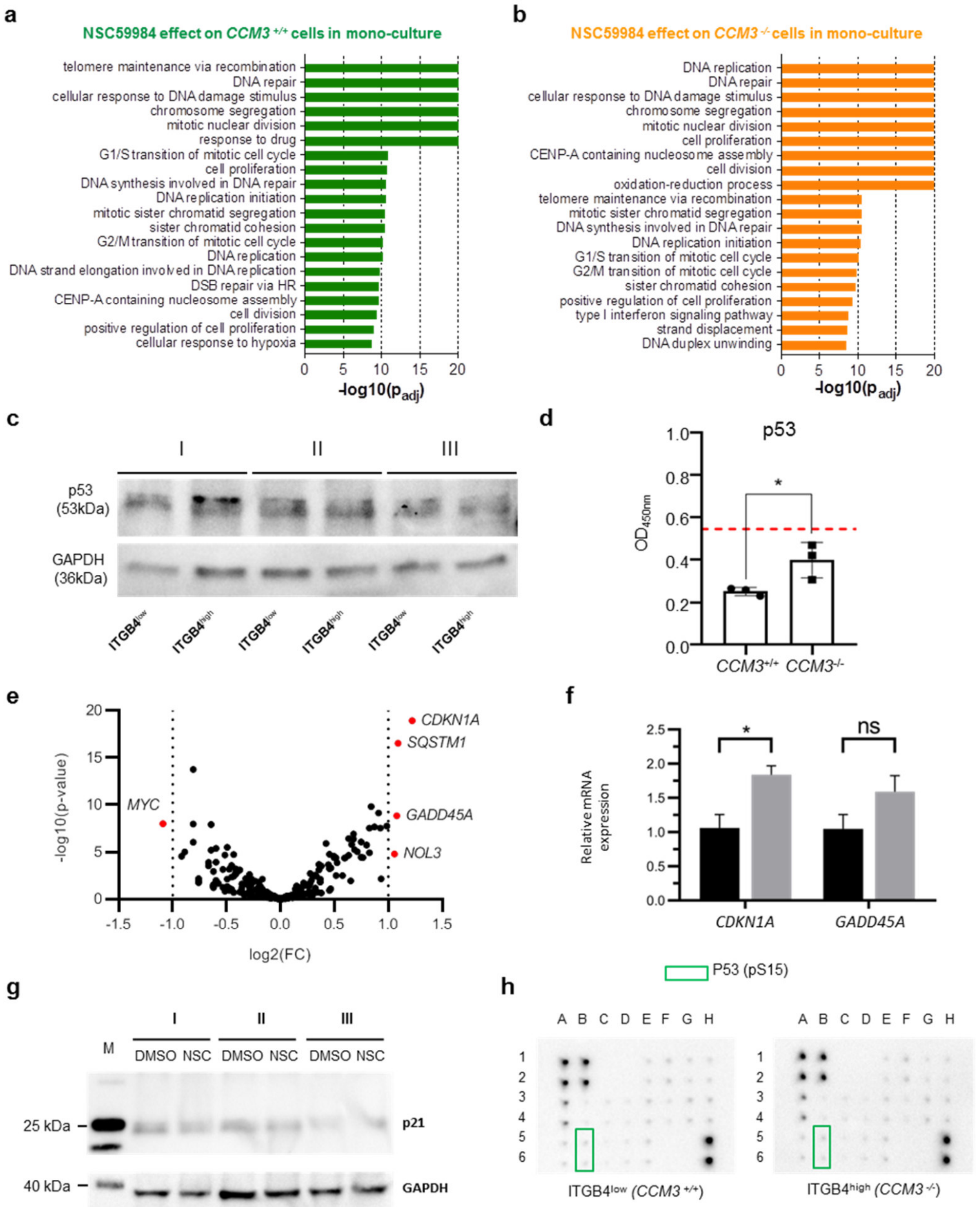

Online Resource 10 Effect of NSC59984 on *CCM3*$^{-/-}$ and *CCM3*$^{+/+}$ CI-huVECs in mono- and co-culture. **a, b** NSC59984-treated *CCM3*$^{-/-}$ and *CCM3*$^{+/+}$ CI-huVEC mono-cultures were analyzed by RNA sequencing. Significantly up- or downregulated genes were subjected to gene ontology analyses. Shown are the top 20 of significantly enriched biological process GO terms (n = 3 per group). **c, d** p53 protein levels and transcription factor binding activities were analyzed in sorted ITGB4^{high} CCM3^{neg} and ITGB4^{low} CCM3^{pos} CI-huVEC populations. The Red line (d) indicates the value of the kit-provided transcription factor p53 positive control. **e** NSC59984-treated *CCM3*$^{-/-}$ CI-huVECs were subjected to targeted RNA panel sequencing (QIAseq targeted RNA human apoptosis

and cell death panel). The results are depicted as volcano plot. FC = fold change (NSC59984 vs. DMSO treatment). Transcripts with a $|Log_2 FC| \geq 1$ and an adjusted p-value < 0.05 are highlighted in red. **f, g** Upregulation of *CDKN1A* (p21) was verified on RNA (f) but not on protein level (g). black bar = DMSO control, grey bar = NSC59984 treatment (n = 3 per group). **h** A MAPK phosphorylation antibody array was used to study MAPK/ERK signaling in sorted ITGB4^{high} CCM3^{neg} and ITGB4^{low} CCM3^{pos} CI-huVEC populations (n = 3 per genotype). Representative array membranes are shown for both genotypes. Green rectangles mark phosphorylated p53 (pS15). No significant differences were found for any markers. Fisher exact test (a, b), Students two-tailed *t* test (d, f), and DESeq2 normalization method (e) were used for statistical analyses: *P<0.05
